# Single-molecule super-resolution imaging of chromosomes and *in situ* haplotype visualization using Oligopaint FISH probes

**DOI:** 10.1038/ncomms8147

**Published:** 2015-05-12

**Authors:** Brian J. Beliveau, Alistair N. Boettiger, Maier S. Avendaño, Ralf Jungmann, Ruth B. McCole, Eric F. Joyce, Caroline Kim-Kiselak, Frédéric Bantignies, Chamith Y. Fonseka, Jelena Erceg, Mohammed A. Hannan, Hien G. Hoang, David Colognori, Jeannie T. Lee, William M. Shih, Peng Yin, Xiaowei Zhuang, Chao-ting Wu

**Affiliations:** 1 Department of Genetics, Harvard Medical School, Boston, Massachusetts 02115, USA; 2 Department of Chemistry and Chemical Biology, Harvard University, Cambridge, Massachusetts 02138, USA; 3 Howard Hughes Medical Institute, Cambridge, Massachusetts 02138, USA; 4 Wyss Institute for Biologically Inspired Engineering, Harvard University, Boston, Massachusetts 02115, USA; 5 Department of Systems Biology, Harvard Medical School, Boston, Massachusetts 02115, USA; 6 Institut de Génétique Humaine, CNRS UPR 1142, 141 rue de la Cardonille, 34396 Montpellier, France; 7 Howard Hughes Medical Institute, Boston, Massachusetts 02114, USA; 8 Department of Molecular Biology, Massachusetts General Hospital, Boston, Massachusetts 02114, USA; 9 Department of Biological Chemistry and Molecular Pharmacology, Harvard Medical School, Boston, Massachusetts 02115, USA; 10 Department of Cancer Biology, Dana-Farber Cancer Institute, Boston, Massachusetts 02215, USA; 11 Department of Physics, Harvard University, Cambridge, Massachusetts 02138, USA

## Abstract

Fluorescence *in situ* hybridization (FISH) is a powerful single-cell technique for studying nuclear structure and organization. Here we report two advances in FISH-based imaging. We first describe the *in situ* visualization of single-copy regions of the genome using two single-molecule super-resolution methodologies. We then introduce a robust and reliable system that harnesses single-nucleotide polymorphisms (SNPs) to visually distinguish the maternal and paternal homologous chromosomes in mammalian and insect systems. Both of these new technologies are enabled by renewable, bioinformatically designed, oligonucleotide-based Oligopaint probes, which we augment with a strategy that uses secondary oligonucleotides (oligos) to produce and enhance fluorescent signals. These advances should substantially expand the capability to query parent-of-origin-specific chromosome positioning and gene expression on a cell-by-cell basis.

## Abstract (web summary)

The spatial organization of the genome within the nucleus impacts many processes. Here the authors combine oligo-based DNA FISH with single-molecule super-resolution microscopy to image single-copy genomic regions and, taking advantage of SNPs, distinguish allelic regions of homologous chromosomes.

## Graphical abstract

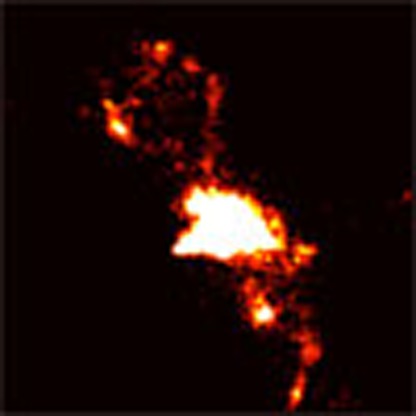

Since their inception^1^,^2^,^3^, *in situ* hybridization techniques have provided critical insights into the spatial organization of nucleic acids within the cell. This family of methodologies has led to the discovery that the eukaryotic nucleus is a highly ordered compartment, with chromosomes falling into distinct territories^4^. Yet, despite decades of advances in hybridization-based single-cell imaging technology, our ability to directly visualize the fine-scale structure of the genome *in situ* remains constrained by the optical resolution of light microscopy and the limitations of our ability to target regions of interest. Consequently, many gaps remain in our understanding of how local chromatin structure and nuclear positioning impact processes such as transcription, the establishment of chromosome–chromosome interactions and DNA repair.

Here we report two strategies for *in situ* single-cell imaging, one that facilitates two forms of single-molecule super-resolution microscopy and another that utilizes SNPs to visually distinguish homologous chromosomal regions. Both make use of Oligopaints, which are highly efficient, renewable, strand-specific fluorescence *in situ* hybridization (FISH) probes derived from complex single-stranded DNA (ssDNA) libraries in which each oligo carries a short stretch of homology to the genome (Fig. 1a). In contrast to classical FISH probes, which are produced from segments of purified genomic DNA amplified in bacterial vectors or PCR reactions, Oligopaints belong to a new generation of probes that are derived entirely from synthetic DNA oligonucleotides (oligos)^5^,^6^,^7^. Such probes have their sequences chosen bioinformatically; thus, they can be designed to target any organism whose genome has been sequenced, engineered to avoid repetitive elements, and selected to have specific hybridization properties. Our studies take advantage of two features of Oligopaints: the inclusion of non-genomic sequences, which enable super-resolution imaging, and a programmable insert of genomic homology, which makes it possible for Oligopaints to bind specifically at SNPs.

## Results

### Implementing secondary oligos

**Figure 1:**
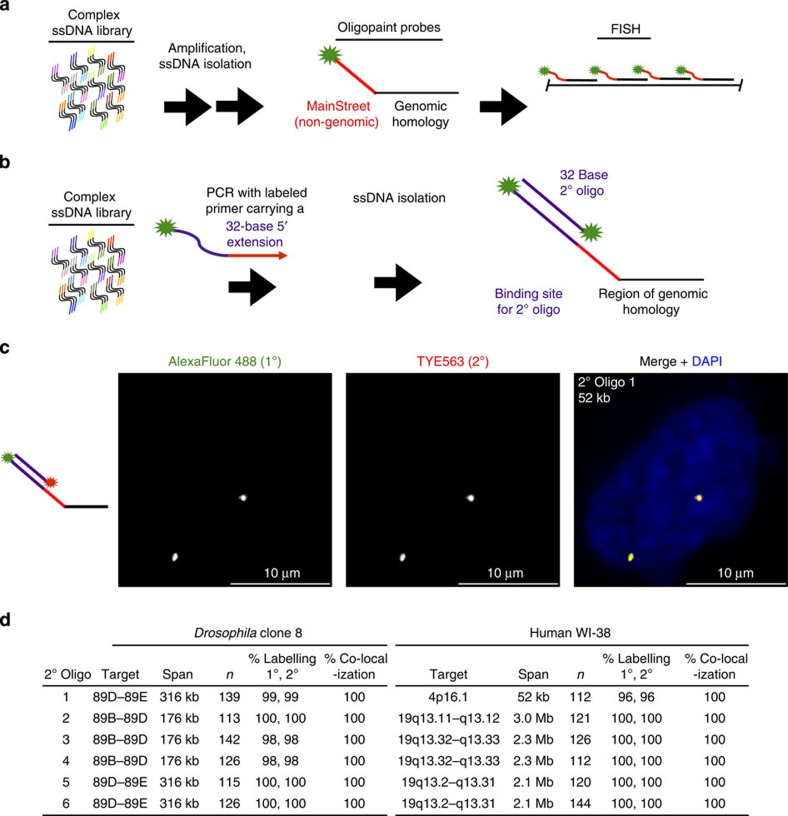
Secondary oligos are specific and efficient. (**a**) One synthesis strategy for Oligopaints, in which complex ssDNA libraries consisting of a stretch of genomic sequence (black lines) on the order of tens of bases flanked by non-genomic regions (coloured lines) containing primer sequences are amplified, labelled and then processed in any of a variety of ways to produce ssDNA probes that carry non-genomic sequences at one (shown) or both (not shown) ends (adapted from ref. 7; also see Supplementary Figs 2 and 3 for more details on MainStreet incorporation and placement strategies). The primer sequence can constitute the entirety, or just a portion, of the non-genomic region, called MainStreet, which will remain single-stranded when Oligopaint probes are hybridized to their target. (**b**) A binding site for a secondary (2°) oligo probe can be introduced to MainStreet by PCR amplification with a primer that carries the binding site. Here, the secondary oligo carries a single, 5′ fluorophore that matches the fluorophore present on the Oligopaint (primary) probe, but in practice the number, identity and placement of fluorophores on the secondary oligo can vary. Also see Supplementary Figs 2 and 3. (**c**) Grayscale and multicolour images from a two-colour co-localization experiment in diploid human WI-38 cells. DNA is stained with 4′,6-diamidino-2-phenylindole (DAPI; blue). Images are maximum *Z* projections from a laser scanning confocal microscope. (**d**) Two-colour co-localization experiments in diploid *Drosophila* clone 8 cells and WI-38 cells. The genomic target, span of the target, number of nuclei examined (*n*), per cent of nuclei (% Labelling) that had at least one signal from the primary (1°, Oligopaint) probe and at least one signal from the secondary oligo and per cent primary signals that have an overlapping secondary signal (% Co-localization) are given for each experiment.

Central to the design of Oligopaints is the inclusion of non-genomic sequences flanking the region of homology to the genome, as these sequences enable the amplification by PCR or other methods to produce DNA or RNA oligos, introduction of label and conversion of double-stranded to single-stranded products^7^ (Fig. 1a). This design also permits the multiplexing of Oligopaint libraries, wherein a single library is used to produce multiple distinct probe sets, each derived from a subset of oligos through amplification via a primer pair specific for that subset^7^ (Supplementary Fig. 1). Furthermore, as a non-genomic sequence is designed to remain single-stranded when Oligopaint probes are hybridized to their genomic targets, it could be used to recruit activities without disruption of targeting. Indeed, the non-genomic sequence, which we call MainStreet^7^, could be populated by any number of functionalities via the binding of complementary oligos, nucleic acid binding proteins or other factors.

We began our current studies by examining the ability of MainStreet to recruit a fluorescently labelled ‘secondary’ oligo, as we were intrigued by the potential of secondary oligos to simplify the use of multiplexed Oligopaint libraries. For example, inclusion of a common binding site for a secondary oligo in the MainStreet of all of the probe sets of a multiplexed library would not only permit all the probe sets to be indirectly labelled *in situ* through the binding of labelled complementary secondary oligos, but would also make a single species of labelled secondary oligo compatible with all the probe sets. Such a strategy would obviate the need to incorporate fluorophores directly into the Oligopaint probes and thereby reduce the number and, hence, cost of fluorophore-labelled oligos needed to utilize heavily multiplexed libraries.

Figure 1b–d illustrates our strategy for testing the potential of secondary oligos. We first used a database of orthogonal sequences^8^ to design six 32-base DNA oligos with thermodynamic properties predicted to be optimal for hybridization in the conditions of our FISH protocols (Supplementary Table 1). Then, using touch-up PCR^9^, we placed a binding site for one or more of the secondary oligos 5′ of the primer sequences in MainStreet (Fig. 1b); this strategy allows binding sites to be added to any existing Oligopaint library and is compatible with both our published probe synthesis protocols^7^,^10^ (Supplementary Figs 1 and 2, Methods) as well as alternative methods for generating Oligopaints, such as our 1-day method using lambda exonuclease^11^,^12^ (Supplementary Fig. 3) and the MYtags strategy (MYcroarray). Binding sites for secondary oligos can also be incorporated during the original design of the library, in which case they could be internal to the primer sequences, with two designs worth considering for multiplexed libraries. In the first, all probe sets, each with its own primer sequences, would carry a common binding site for secondary oligos, permitting researchers to use a common labelled secondary oligo to image all probe sets. In the second design, all probe sets would carry common primer sequences but distinct binding sites for distinct secondary oligos, enabling researchers to amplify all probe sets simultaneously and then separately image each probe set with distinct labelled secondary oligos.

To assess the effectiveness of secondary oligos, we conducted two-colour co-localization experiments in *Drosophila* and human cell culture. In these experiments, Oligopaint probe sets targeting regions ranging in size from 52 kb to 3 Mb and consisting of hundreds to tens of thousands of oligos, each bearing 32 or 42 bases of homology to the genome (Supplementary Table 2, Supplementary Note 1) and a 5′ fluorophore as well as a binding site for a secondary oligo, were co-hybridized with a secondary oligo carrying a spectrally distinct fluorophore. We found all six of our secondary oligos to be remarkably specific, with 100% of the signals coming from the secondary oligos co-localizing tightly with the signals of the primary Oligopaint probes in both Drosophila diploid clone 8 and human diploid WI-38 cells (*n*>100 for all cases; Fig. 1c,d and Supplementary Fig. 4; 177 nm chromatic between the red and green channels factored into determination of % Co-localization (see Methods and Supplementary Fig. 5). The two-colour FISH was also efficient; 96–100% of nuclei (*n*>100 for all cases) displayed signals (Fig. 1d), with diploid human cells showing primarily two sets of co-localized signals, while diploid *Drosophila* cells, which pair homologous chromosomes in somatic cells^13^, showing primarily single sets of co-localized signals representing both the maternal and paternal copies of the targeted region. The secondary oligos can be added simultaneously (Fig. 1c,d) or sequentially (Supplementary Fig. 6) and produce only weak speckling when they are added in the absence of primary probes. We observed a similarly robust performance when using 14-base secondary oligos containing locked nucleic acid (LNA)^14^ residues (Supplementary Table 1). Here we used a single synthetic oligo, carrying a 32-base MainStreet and targeting the highly repetitive 359 satellite sequences on the *Drosophila* X chromosome in clone 8 cells (100% co-localization, >99% efficiency for each of 3 LNAs, *n*>100 in all cases, Supplementary Fig. 7); these LNA secondary oligos can either be directly labelled or programmed to form branched structures that amplify signals^15^ (Supplementary Figs 8–10). In sum, our results suggest that secondary oligos hybridize efficiently to MainStreet and do not hinder the ability of Oligopaint probes to associate with their genomic targets, suggesting that MainStreet could also be used to augment the number of fluorophores at a genomic target via the recruitment of multiple secondary oligos, enable the combinatorial use of different fluorophores, and support applications involving Förster resonance energy transfer^16^ (Supplementary Fig. 11).

### Enabling super-resolution FISH with Oligopaints

**Figure 2:**
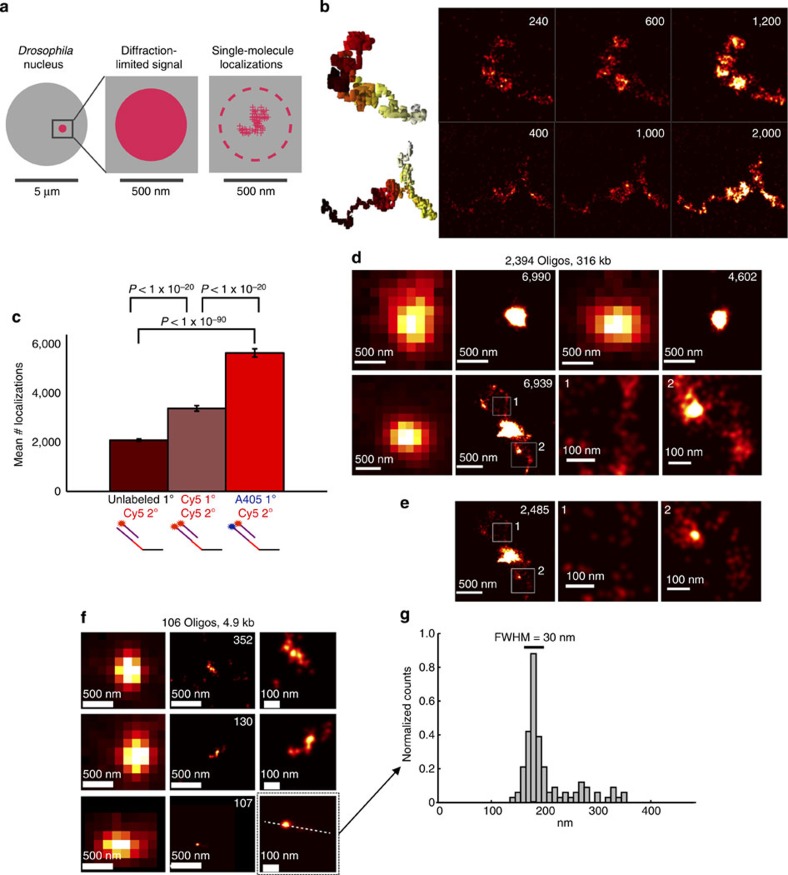
Super-resolution imaging with Oligopaints and STORM. (**a**) Schematic illustrating how a diffraction-limited FISH signal presents as many smaller fluorescence localizations via STORM. (**b**) Simulated STORM images of two polymer models (left) illustrating the importance of localization density in resolving structure (total localizations in upper right corners). The colour code on the polymer models traces along the length of the polymer (black to red to white). (**c**) Average number of localizations (mean±s.e.m.; *n*=434 for unlabeled/Cy5, *n*=133 for Cy5/Cy5, *n*=353 A405/Cy5) per BX-C locus in *Drosophila* clone 8 cells when the unlabelled primary probe is paired with a secondary oligo carrying Cy5 (left), when both the primary probe and secondary oligo carry Cy5 (middle), and when the primary probe carrying an AlexaFluor 405 activator is paired with a secondary oligo carrying Cy5. (**d**) Conventional (left) and STORM (right) images of the BX-C locus from three cells, with cell shown in bottom row exhibiting two loop-like protrusions. The conventional and STORM images depict the same field of view at the same magnification. Right two panels: zoomed-in views of the boxed regions. (**e**) Simulation in which two-thirds of the localizations shown in image (**d**) have been removed at random to illustrate the loss of connectivity and structure in regions represented by a low density of localizations. (**f**) Conventional (left) and STORM (middle and right) images of a 5-kb region at 89B from three cells. Right panel: zoomed-in views of the centre panels. (**g**) A graph of the normalized number of photons detected (Normalized counts) per position (nm) in the *x* axis (dashed line) of the field shown in the bottom-right panel of **f**. The FWHM of the brightest feature is presented above the graph. Super-resolution images are presented as heat maps of single-mole localization density: black (fewest) -> red -> yellow -> white (most).

The efficacy of secondary oligos raised the potential of their application for super-resolution microscopy^17^,^18^,^19^. As diffraction limits the resolution of conventional light microscopy to a distance of ∼200 nm in the *x*–*y* plane and ∼500 nm in the *z* direction, the volume of a diffraction-limited signal is considerably larger than that of many nuclear structures. Researchers can overcome this diffraction-limited resolution, however, through the use of super-resolution imaging technologies (Fig. 2a). Structured illumination microscopy^20^,^21^ has been the most broadly used super-resolution method to date for imaging genomic loci *in situ*^22^,^23^,^24^,^25^,^26^,^27^. Here we explore a different family of super-resolution technologies, which rely on stochastically occurring single-molecule fluorescence events to localize the position of each fluorophore molecule with high precision. These single-molecule-based super-resolution techniques can enhance our understanding of nanoscale structural features, as their resolution is limited only by the number of photons collected per fluorophore and the density at which the target structure is labelled with fluorophores^18^.

Excitingly, a few studies have used single-molecule approaches to image chromosomes *in situ*. In one study, a single peptide nucleic acid oligo probe was used to visualize repetitive sequences at the centromere of human chromosome 9 with localization precisions as low as 10–20 nm, thus resulting in an obtainable resolution of ∼25–50 nm (full width at half maximum, FWHM)^28^. Another study used a fragments of DNA derived from the DYZ2 repeat to visualize heterochromatin on the human Y chromosome with an average resolution of ∼50 nm (FWHM)^29^. A third study used a single peptide nucleic acid probe to visualize repetitive telomeric sequences on spread mouse chromosomes with ∼20 nm resolution (FWHM)^30^. Of note, however, is that all these studies targeted repetitive regions of the genome, where high copy numbers of the tandemly repeated target sequences allowed for dense labelling using single oligo species or a short insert of cloned genomic DNA as the source of FISH probe.

Given the ease with which Oligopaint probe sets can be designed and generated, we predicted that they would render single-copy genomic regions and regions consisting of repeated sequences equally amenable to single-molecule super-resolution imaging. Furthermore, Oligopaints could enhance the interpretation of super-resolution images, as they afford direct control over the number, position and placement of fluorophore molecules on each Oligopaint oligo as well as those on any secondary oligos hybridized to MainStreet. Finally, we reasoned that our ability to control the length, orientation and positioning of secondary oligos along MainStreet would allow for the reliable placement of the fluorescent signal directly at the site of hybridization (Supplementary Fig. 11), making them an ideal tool for tracing genomic structure at high resolution. This in mind, we first set out to explore the potential of combining Oligopaint probes with stochastic optical reconstruction microscopy (STORM)^31^, which relies on the stochastic activation and localization of individual photoswitchable fluorophores to produce super-resolution images^32^.

In this study, we used the photoswitchable cyanine dye Cy5 for STORM imaging. Cy5 can exist in two states—a ‘bright’ state, where it emits fluorescence on excitation, and a ‘dark’ state, where it is not capable of fluorescing. Importantly, activation of Cy5 from the dark to the bright state can be enhanced by a nearby ‘activator’ dye. For instance, use of AlexaFluor 405 as the activator dye allows for photoswitching to be induced with an intensity of 405 laser excitation that is lower than that which would be used in the absence of activator dye, thus keeping the rate of 405 nm light-induced photobleaching low. In such an instance, more localizations can be recorded, thus improving the sampling resolution of the image. To explore the potential effects of localization density on resolution for chromatin structures, we simulated STORM images from hypothetical polymer structures (Fig. 2b). We found that simulations with a low number of total localizations appeared more frequently as disconnected objects; while densely coiled parts of the polymer appeared similar across a broad range of total localizations, long protrusions and narrow bridges became difficult to distinguish from low levels of background when the number of total localizations was small.

We next harnessed our ability to create precise fluorophore–fluorophore pairings with Oligopaints and secondary oligos (Supplementary Fig. 11), targeting 2,394 Oligopaint oligos to the developmentally regulated 316 kb bithorax complex (BX-C)^33^,^34^,^35^ in diploid *Drosophila* clone 8 cells for visualization. In particular, we paired Cy5-labelled secondary oligos with a primary probe set that carried either no label, a Cy5 or an AlexaFluor 405. Excitingly, all three primary–secondary pairings were able to produce super-resolution FISH images (Supplementary Fig. 12). While all three primary–secondary pairings were effective, we observed a significantly greater number of single-molecule localizations when an AlexaFluor 405 activator dye was paired with the Cy5 reporter (median±s.e.m: 2,075±49, *n*=434 for unlabelled primary/Cy5-labelled secondary; 3,364±114, *n*=133 for Cy5/Cy5; 5,612±167, *n*=353 for A405/Cy5; Fig. 2c, Supplementary Fig. 12), demonstrating the effectiveness of dye pairing enabled by secondary oligos. The less than double the number of localizations observed with two Cy5 dyes per probe as versus a single Cy5 dye per probe is likely the result of quenching interactions between the reporter dyes. By taking advantage of the higher density of localizations made possible through the activator–reporter labelling strategy, we detected fine-scale nanostructures of chromatin, such as the one shown in Fig. 2d, which is not visible in the diffraction-limited image of the same field. Indeed, while we find the BX-C locus in most cells to lack substantial protrusions, we did occasionally observe threads of chromatin appearing to loop away from the primary cluster of signals. Importantly, we found that if we approximate the labelling density obtained with a single Cy5 dye by removing two-thirds of localizations from our images at random, the shapes of the protrusions are not as clear (Fig. 2e), with some segments becoming more difficult to distinguish from background (Supplementary Fig. 12). Our activator–reporter system also allowed us to examine a much smaller genomic region. In this case, we targeted 4.9 kb at chromosome position 89B in tetraploid *Drosophila* Kc_167_ cells with 106 Oligopaint oligos and produced super-resolution images displaying intriguing morphologies (Fig. 2f), including structural features <35 nm in size (Fig. 2g).

**Figure 3:**
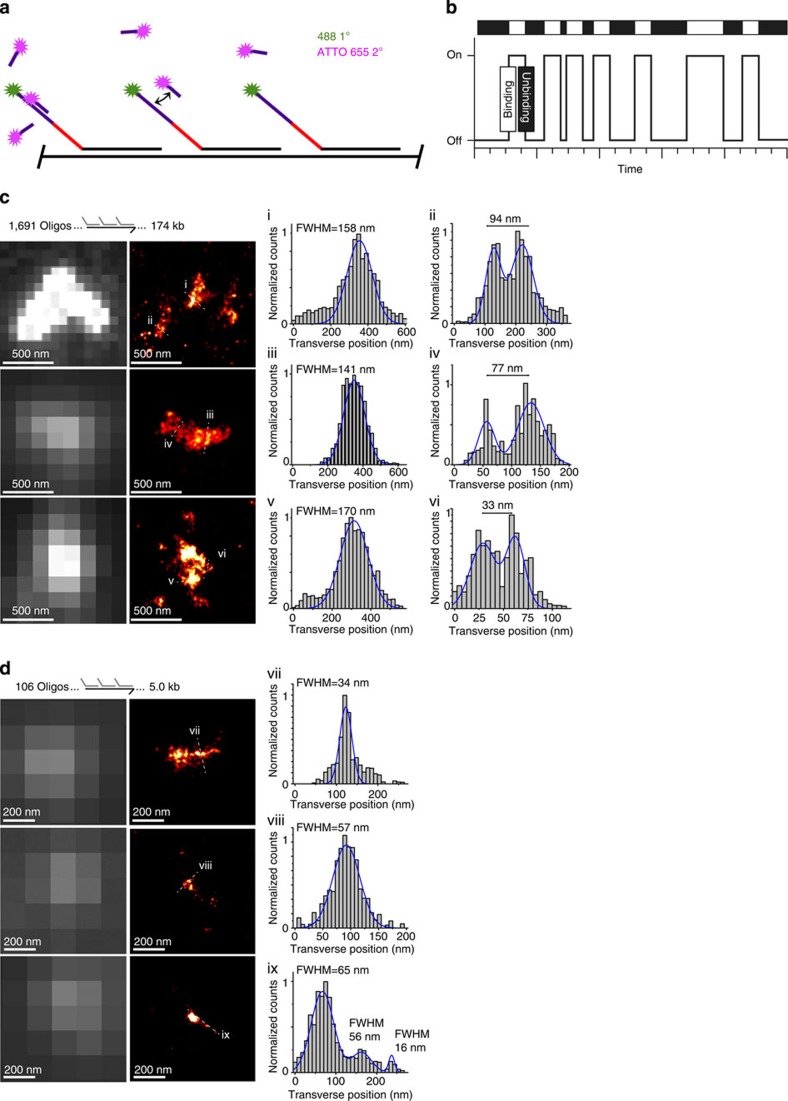
Super-resolution imaging with Oligopaints and DNA-PAINT. (**a**) Labelling scheme using Oligopaint probes carrying an ATTO 488 dye and a 9-base docking site that is complementary to imager strands labelled with ATTO 655. (**b**) Trace of Intensity versus time showing the transient binding of imager strands and docking strands or ‘blinks’. (**c**,**d**) Diffraction-limited images obtained with ATTO 488 (left) and DNA-PAINT super-resolution images obtained with ATTO 655-labelled imager strands at 5 nM (right) of Oligopaint probe sets labelled with ATTO 488 and targeting 174 kb (**c**) and 5 kb (**d**) of the mouse hoxB locus in MEFs. To the right of the images are cross-sectional (dotted lines in DNA-PAINT images i–ix) histograms displaying the normalized number of photons detected (normalized counts) versus transverse position for each region. Structural features are inferred from these transverses with one-dimensional Gaussian fits, with FWHMs indicated above each graph. Imaging: 15,000 frames at 10 Hz rate. Super-resolution images are presented as heat maps of single-mole localization density: black (fewest) -> red -> yellow -> white (most).

We also explored the potential of Oligopaint primary–secondary pairings to enable the visualization of single-copy genomic regions using a related single-molecule-based super-resolution approach called DNA-based point accumulation for imaging in nanoscale topography (DNA-PAINT)^36^,^37^,^38^. In DNA-PAINT, the single-molecule fluorescence events are generated by the transient hybridization of fluorescently labelled oligonucleotides, called ‘imager strands’, present in solution in the imaging buffer to complementary strands, called ‘docking strands’, on the target to be imaged, reminiscent of the binding of secondary oligos to the MainStreet of Oligopaints (Fig. 3a); as the duplexes that form are designed to be unstable at room temperature (RT, duplex length of 9 bases; bound time in imaging conditions ≈600 ms (ref. 37), the transient binding interactions lead to an apparent ‘blinking’ of the docking sites when imaged using configurations, such as total internal reflection fluorescence (TIRF) microscopy or highly inclined and laminated optical sheet^39^ microscopy, which provide high ratios of signal:noise (Fig. 3b).

To explore the feasibility of enabling DNA-PAINT imaging of chromosomes with Oligopaints, we designed a probe set consisting of 1,691 oligos carrying a binding site for an imager strand carrying an ATTO 655 fluorophore and targeting the developmentally regulated 174 kb hoxB locus^35^ in mouse. Application of this probe set to transformed mouse embryonic fibroblasts (MEFs; Fig. 3c) produced super-resolution images, wherein we were able to visualize nanoscale structural features at this locus <50 nm in size (Fig. 3c). Importantly, we were able to maintain a constant number of single-molecule localizations per frame over the entire course of image acquisition because, as imager strands are continuously replenished from solution, photobleaching does not present a significant problem for DNA-PAINT (Supplementary Fig. 13). Indeed, we were able to harness this feature to produce super-resolution images of a 5-kb portion of the hoxB cluster using a probe set consisting of only 106 oligos, wherein our sampling capacity allowed us to resolve structural features as small as 16 nm (Fig. 3d).

Together these single-molecule super-resolution imaging results demonstrate that Oligopaints are a powerful tool for visualizing single-copy genomic loci. Given the high image resolutions achieved here, it is worth noting, nevertheless, that the biological relevance of the structures we have observed will only become apparent after extensive application of our technologies under a variety of laboratory settings enables us to evaluate to what extent the structures observed are affected by the experimental conditions of FISH labelling.

### Distinguishing homologous chromosomes with Oligopaints

While the methods described above can enhance our capacity to resolve chromosomal structures, they do not address one of the most intractable challenges in single-cell studies of chromosome positioning and gene expression, which is the visual distinction of maternal, paternal and, indeed, any homologous chromosomes (homologues). Strategies for distinguishing homologous chromosomes and chromosomal regions would greatly advance our capacity to investigate phenomena such as X-inactivation^40^, imprinted gene expression^41^ and random monoallelic expression^42^; the few methods that are available either rely on relatively inefficient enzymatic signal amplification strategies^43^,^44^,^45^ or are appropriate only for highly repetitive portions of the genome^46^ or RNA molecules^47^,^48^,^49^, and thus cannot be used to visualize single-copy regions or loci that are not expressed in the sample of interest. We have addressed this challenge by developing homologue-specific OligoPaints, or ‘HOPs.’

HOPs take advantage of the abundant and well-characterized single-nucleotide polymorphism (SNP) data, such as those provided by the Wellcome Trust Sanger Mouse Genomes Project^50^ and the Drosophila Genetic Reference Panel (DGRP)^51^. In our approach, we first generate short blocks of reference genomic sequence centred on each SNP in the region we wish the HOPs to target (Supplementary Fig. 14). We then input these blocks into our Oligopaint probe discovery pipeline^7^ to identify probe sequences that overlap the location of at least one SNP, are genomically unique, and have suitable thermodynamic properties. Finally, we run a custom Python script to insert the SNP variant(s) into the probe sequences. Importantly, HOP probe sets are always made in pairs; that is, each oligo of a HOP probe set has a cognate oligo in its partner probe set, where both oligos span precisely the same genomic coordinates and differ only by the SNP variant(s) they carry. Thus, partner HOP probe sets target the same region on different homologues by utilizing differences in the haplotypes of these chromosomes.

**Figure 4:**
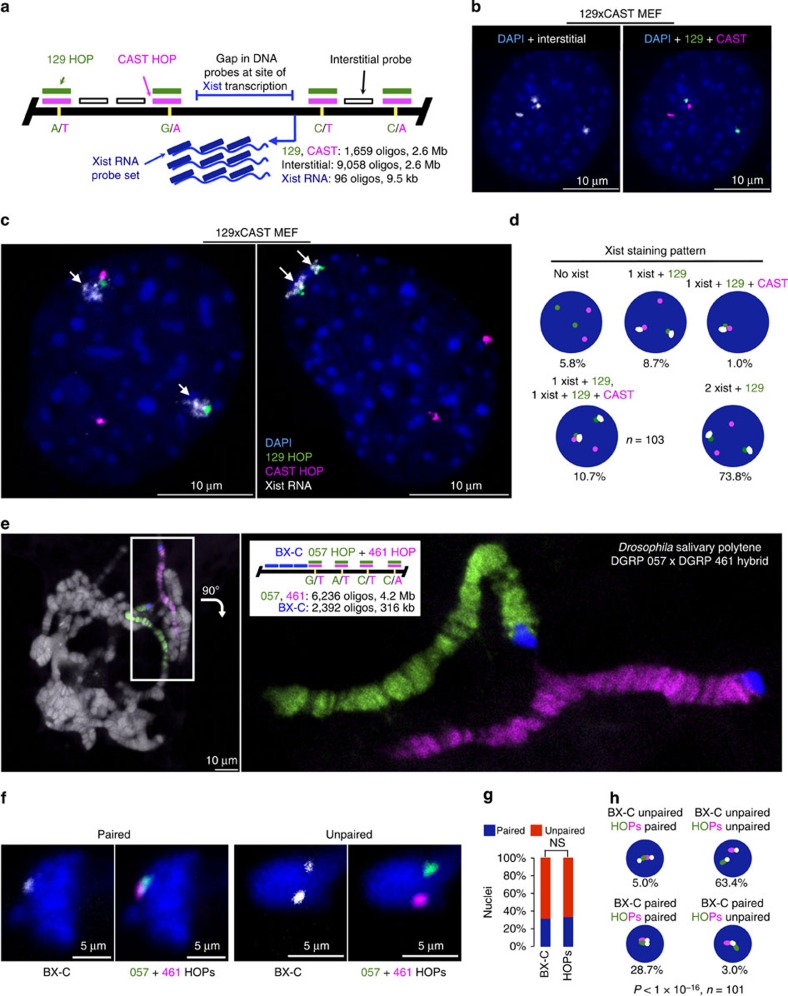
HOPs. (**a**) Schematic of HOPs targeting the mouse XIC (not to scale). 129 (green) and CAST (magenta) HOPs are targeted to SNPs and carry variants specific for the 129S1/SvImJ (129) or CAST/EiJ (CAST) genomes, respectively, while interstitial (white) probes target sequences common to both genomes. None of these three probe sets target the Xist transcript, which is targeted by a fourth Oligopaint probe set (blue) (**b**) Hybrid EY.T4 129xCAST-transformed MEF cells visualized with 129 (green) and Cast (magenta) HOPs and the interstitial probe set (white). The interstitial probe set binds 129 and CAST chromosomes equally well (left), while the 129 and CAST HOPs reveal the parent-of-origin of the interstitial signals (right). (**c**) RNA/DNA FISH with 129 (green) and CAST (magenta) HOPs and Xist RNA FISH (white) demonstrating co-localization of Xist signal with that of the 129 HOP. Arrows point to Xist signals. (**d**) Percentage of nuclei falling into each of five Xist staining patterns. (**e**) Polytene chromosomes of a *Drosophila* salivary gland nucleus (left) and enlarged image of boxed region (right) from DGRP 057 × DGRP 461 hybrid larvae visualized with Oligopaints targeting the BX-C (blue) and 057-specific (green) and 461-specific (magenta) HOPs targeting the flanking 89E–93C region. DNA is stained with 4′,6-diamidino-2-phenylindole (DAPI; grey), which is removed from right image. Images are single *Z* slices from a laser scanning confocal microscope. (**f**) *Drosophila* 6–8 h embryo nuclei visualized with the BX-C probe set (white) and the 057 (green) and 461 (magenta) HOPs showing the paired (left) and unpaired (right) at both BX-C and the adjacent 89E–93C region. (**g**) % Pairing observed at BX-C and 89E–93C, where loci were considered paired if edge-to-edge distance between their signals was ≤0.8 μm. (NS, not significant, two-tailed Fisher’s exact *P*=0.88, *n*=101). (**h**) The paired status of BX-C is statistically associated with that of 89E–93C (two-tailed Fisher’s exact *P*=6.4 × 10^−17^, *n*=101). For **b**, **c**, and **f**: DNA is stained with DAPI (blue). Images are maximum *Z* projections from a laser scanning confocal microscope.

In our first test of the HOPs system, we examined a 2.6-Mb region containing the murine X-inactivation centre (XIC), which produces the Xist RNA^40^, in three SV-40 large T-antigen-immortalized MEF lines (Fig. 4a). These lines, all of which appear to carry four copies of the X chromosome, are derived from three strains of mice: 129S1/SvImJ (129), CAST/EiJ (CAST) and hybrid 129xCAST mice^52^. Importantly, the 129 and CAST genomes differ by an average of two to three SNPs per kb both in the 2.6-Mb region of the XIC and across the entire genome, and, furthermore, our HOP probe discovery pipeline determined that ∼40% of the SNPs occurred in genomic sequences suitable to serve as an Oligopaint FISH probe. This density of variants allowed us to design 129-specific and CAST-specific sets of HOP probes targeting the XIC region, each of which consisted of 1,659 oligos. We also designed 9,058 ‘interstitial’ probes that target the same 2.6-Mb XIC region but avoid all SNPs and HOPs and thus should bind both 129 and CAST chromosomes equally well. All three probe sets also avoided the genomic region from which Xist is transcribed, thus giving us the option to perform simultaneous RNA/DNA FISH^7^ by including a fourth probe set consisting of 96 oligos targeting the Xist RNA.

We first simultaneously hybridized AlexaFluor 488-labelled 129 HOP (green), ATTO 565-labelled CAST HOP (magenta) and ATTO 633-labelled interstitial probes (white) to the three aforementioned MEF lines. As expected, the interstitial probes produced strong staining in all three lines (Supplementary Fig. 15). A notably different, homologue-specific staining pattern was observed with the HOP probe sets (Fig. 4b). Specifically, the signals of each HOP co-localized with approximately half of the interstitial probe signals in hybrid EY.T4 129xCAST MEFs (49.5 and 50.5% of interstitial probe signals co-localized with 129 and CAST HOP signals, respectively; *n*=111 nuclei, 440 signals; Supplementary Fig. 15), 100% of the 129 HOP signals co-localized with the interstitial probe signals in 129 MEFs (*n*=111 nuclei, 401 signals) and 100% of the CAST HOP signals co-localized with the interstitial probe signals in CAST MEFs (*n*=111 nuclei, 452 signals). The homologue-specific staining was highly efficient, with 100% of nuclei displaying signals in all three cell types. It was also robust to differences in the relative concentrations of the two HOPs (Supplementary Fig. 16) but likely dependent on competition between the HOPs, as the addition of either HOP alone resulted in the HOP signal co-localizing with 100% of the interstitial signals in 129xCAST MEFs (*n*≥57 nuclei, 190 signals in both cases; Supplementary Fig. 16).

We then confirmed the specificity of HOPs by taking advantage of the fact that the EY.T4 129xCAST MEF line, which is female, has a pattern of X-inactivation in which the X_CAST_ is always the active X chromosome (Xa), and the X_129_ is always the inactivate X chromosome (Xi)^52^. Because of this pattern, the X_129_ is expected to be coated in *cis* with the Xist RNA^40^ and thus presents an independent means by which to visually identify the *in situ* position of the X_129_ chromosome. Accordingly, we performed simultaneous RNA/DNA FISH by using the XIC HOPs in conjunction with an Oligopaint probe set consisting of 96 oligos targeting a 9.5-kb portion of the Xist RNA and observed the tight co-localization of 100% of Xist signals (*n*=101 nuclei, 183 signals) with signals of the 129 HOP (Fig. 4c,d and Supplementary Fig. 17). In contrast, the Xist signal rarely co-localized with the CAST HOP (6.5% of 183 Xist signals) and only did so when a 129 HOP signal was also co-localized at the same nuclear position. We also tested smaller sets of HOPs, targeting 998 and 490 kb at the XIC with just 603 and 308 oligos. Again, we observed co-localization of 100% of Xist signals with those of the 129-specific HOPs (*n*=37 nuclei, 52 signals and *n*=38 nuclei, 50 signals, respectively; Supplementary Fig. 18). In addition, quantification of the frequency of ‘crosstalk’ between the partner HOPs, wherein weak staining in the channel for a particular HOP occasionally accompanies a much stronger signal in the channel of its partner HOP, revealed that the smaller sets of HOPs displayed less crosstalk (18.1% for 2.6 Mb, *n*=138 signals; 1.4% for 998 kb, *n*=144 signals; 0% for 490 kb, *n*=132 signals; Supplementary Fig. 18). In sum, our data provide strong evidence that the HOP system can efficiently and reliably distinguish the maternal and paternal homologous chromosomes in the MEF cell culture.

We have also had success with HOPs in *Drosophila*. Here we examined F1 hybrids produced from a cross of the 057 and 461 lines from the DGRP^51^ and targeted a 4.2-Mb region (89E–93C) that is adjacent to the BX-C on the right arm of chromosome 3. This strategy allowed us to use the 2,394 oligo probe set targeting the 316-kb BX-C region (Supplementary Table 2) in lieu of a set of interstitial probes to confirm that our HOPs were localizing properly to their genomic targets (Fig. 4e). Comparing the 89E–93C regions of the 057 and 461 lines, we found approximately seven SNPs per kb, which is somewhat higher than the genome-wide average of approximately five SNPs per kb. We then used our HOP probe discovery pipeline to determine that ∼40% of the SNPs occurred in sequences suitable to serve as Oligopaint FISH probes, of which we selected 6,236 to design a pair of 057-specific and 461-specific HOP probe sets. Excitingly, simultaneous hybridization of the AlexaFluor 488-labelled 057 HOP (green), ATTO 565-labelled 461 HOP (magenta) and ATTO 633-labelled BX-C (blue) probe sets on spread, polytenized chromosomes isolated from the salivary glands of 057/461 hybrid larvae produced a striking pattern of staining in which two swaths of chromosome, both flanked by a blue BX-C signal, were painted either green or magenta (Fig. 4e). This pattern of homologue-specific staining was not observed in polytene chromosomes isolated from the homozygous parental lines (Supplementary Fig. 19). Applying the probes to ovaries, we also found that HOPs are effective in whole-mount tissues (Supplementary Fig. 20).

Just as the X-inactivation pattern of the EY.T4 cell line offered an independent visual assessment of the reliability of HOPs in mammals, the phenomenon of somatic homologue pairing provided a means by which to test the effectiveness of HOPs in *Drosophila*. Traditionally, the state of pairing of a given locus is assayed via FISH, wherein paired homologous loci are predicted to produce a single FISH signal, while unpaired loci are predicted to produce two spatially separated signals. However, if HOPs can reliably distinguish homologous loci *in situ*, we would instead expect two signals in both situations, with the HOP signals being co-localized in the paired state and spatially separated in the unpaired state. To test this idea, we simultaneously hybridized our BX-C probe set (white) and our 057-specific (green) and 461-specific (magenta) HOPs targeting the flanking 89E–93C region to *Drosophila* embryos that were 6–8 h old, when homologue pairing is being established^53^. We observed that the levels of pairing at the BX-C (32% one signal, 68% two signals, 0% no signal, *n*=101; Fig. 4f,g) and the adjacent 89E–93C region (34% co-localized signals, 66% spatially separated signals, 0% no signal, *n*=101; Fig. 4f,g) were not statistically different (Fisher’s two-tailed exact *P*=0.88; Fig. 4g). Importantly, we found the pairing status of these two loci to be highly concordant in individual cells (92.1% concordance with 28.7% both paired and 63.4% both unpaired, Fisher’s two-tailed exact *P*=6.4 × 10^−17^, *n*=101 nuclei from two embryos; Fig. 4h). These results demonstrate that HOPs provide a reliable readout of the individual behaviours of the paternal and maternal homologues.

## Discussion

In sum, we have presented two advances—Oligopaints enabled single-molecule super-resolution imaging of unique genomic regions and HOPs, both of which take advantage of the fully programmable nature of our Oligopaint FISH probes. Together, these tools should enable allele-specific studies of the relationship between gene expression and chromosome organization ranging from overall chromosome positioning to fine-scale chromatin structure, including intra- and interchromosomal interactions. Given the precision at which we have localized single molecules *in situ*, we further anticipate that our technologies will permit the visualization of very short genomic regions, such as those on the scale of enhancers and promoters, with a minimum number of oligo probes. Here studies may benefit from our capacity to engineer Oligopaint oligos to carry a precise number of fluorophores or binding sites for secondary oligos in any number of geometries, thus simplifying the interpretation of fluorescent signals. For example, MainStreet designs that position STORM activator–reporter pairings and DNA-PAINT imager strand-binding sites directly adjacent to the site of genomic hybridization, as versus more distally on MainStreet, would enhance the capacity of our technologies to elucidate fine-scale structures, as minimizing the distance between fluorophores and their genomic target will improve the obtainable structural resolution of the resulting images. Our strategies could also be enhanced through the use of multiple STORM activator–reporter dye pairings^54^, facilitated by secondary oligos, or a highly multiplexed version of DNA-PAINT, called Exchange-PAINT^38^. Finally, we note that since HOPs can produce signals using only one SNP every 1–2 kb, they should be generally applicable, including in humans, where the maternal and paternal genomes differ on average by at least ∼1 SNP per kb^55^,^56^. As such, a combination of HOPs and Oligopaint-facilitated STORM or DNA-PAINT should enable very high resolution, homologue-specific imaging of chromatin structure, with the potential of companion interstitial probes providing even finer-grain information.

## Methods

### Oligonucleotide libraries

The 27E7-28D3, 89D–89E/BX-C, 89B–89D, 4p16.1, 19q13.11–q13.12 and 19q13.2–q13.31 libraries were synthesized by MYcroarray (Ann Arbour, MI). The 19q13.32–q13.33, HoxB, XIC interstitial, XIC HOPs, XIC 490 kb and 998 kb HOPs and 057/461 HOPs libraries were synthesized by CustomArray (Bothell, WA). The 89B 5 kb, HoxB 5 kb and Xist RNA libraries were synthesized by Integrated DNA Technologies (IDT; Coralville, IA). Please see Supplementary Table 2 for a list of Oligopaint probe sets used in this work.

### PCR primers and secondary oligos

Fluorophore-labelled PCR primers, 5′ phosphorylated PCR primers used in the lambda exonuclease protocol, DNA secondary oligos and 359 satellite probe oligos were purchased from IDT and purified by IDT using high-performance liquid chromatography. Unlabelled, unphosphorylated primers were also purchased from IDT and purified by IDT using standard desalting. Fluorophore-labelled LNA/DNA mixers were synthesized by Exiqon (Vedbaek, Denmark) and purified by Exiqon using high-performance liquid chromatography. Please see Supplementary Table 3 for a list of PCR primer pairs and Supplementary Table 4 for a list of secondary oligos used.

### Emulsion PCR amplification of oligonucleotide libraries

Raw, multiplexed libraries purchased from CustomArray (see above) were amplified using universal primers using emulsion PCR to generate template to use in subsequent PCR reactions. Hundred μl of aqueous PCR master mix was gradually mixed into a 600-μl of 95.95% mineral oil (Sigma M5904):4% ABIL EM90 (Degussa):0.05% Triton-X-100 (Sigma T8787) oil phase (v/v/v) at 1,000 r.p.m. for 10 min at 4 °C. Reactions were amplified with the following cycle: 95 °C for 2 min; 30 cycles of 95 °C for 15 s, 60 °C for 15 s and 72 °C for 20 s, with a final extension step at 72 °C for 5 min. After cycling, the DNA was recovered by a series of organic extractions: first using diethyl ether (Sigma 296082), then using ethyl acetate (Sigma 494518); then once again using diethyl ether. These extractions were followed by a phenol–chloroform extraction to remove *Taq* polymerase. For stepwise emulsion, PCR and emulsion-breaking protocols, please see the Oligopaints website ( http://genetics.med.harvard.edu/oligopaints); also see ref. 7.

### Oligopaint probe synthesis

Oligopaints probes containing secondary oligo-binding sites were synthesized using a previous developed gel extraction method or using the lambda exonuclease method introduced here (see below). In either case, the secondary oligo-binding sites were added to Oligopaint probe sets through the use of the following ‘touch-up’ PCR cycle: 95 °C for 5 min; three cycles of 95 °C for 30 s, 60 °C for 45 s and 72 °C for 30 s; 40 cycles of 95 °C for 30 s, 68 °C for 1 min and 72 °C for 30 s, with a final extension step at 72 °C for 5 min. If the probe was produced using the ‘two-PCR’ method (Supplementary Fig. 2), the template generated via ‘touch-up’ PCR was further amplified with the following cycle: 95 °C for 5 min; 40–43 cycles of 95 °C for 30 s, 60 °C for 30 s and 72 °C for 15 s, with a final extension step at 72 °C for 5 min. In the case of the gel extraction method, labelled dsDNA duplexes were digested with Nb.BsrDI (New England Biolabs R0648) and labelled ssDNA probe was isolated by gel extraction from a 10% TBE-urea polyacrylamide gel. See below for details on the lambda exonuclease method. The Xist RNA probe was first extended from 70 to 84 bases in a ‘touch-up’ PCR as before one round of labelling PCR using the ‘touch-up’ cycle described above. One hundred pmol of each primer and 20 pg of template were used per 100 μl of PCR. For stepwise probe synthesis protocols, please see the Oligopaints website ( http://genetics.med.havard.edu/oligopaints); also see refs 7, 10.

### ‘One-day’ probe synthesis using lambda exonuclease

Oligopaint probe sets were amplified using the ‘two-PCR’ method described above, but with the unlabelled primer being phosphorylated on its 5′ end. The PCR reaction was then collected, concentrated using spin columns (Zymo D4031) and digested with lambda exonulcease (New England Biolabs M0262). Five units of lambda exonulcease were added per every 100 μl of unconcentrated PCR reaction (for example, use 50 units if the labelling PCR had a volume of 1 ml before concentration by the spin column) and the reaction was incubated at 37 °C for 30 min in a programmable thermocycler and then stopped by incubation at 75 °C for 10 min. Finally, the digestion products were concentrated using ethanol precipitation and quantified using spectrophotometry. For a detailed protocol, please see the Oligopaints website ( http://genetics.med.havard.edu/oligopaints).

### Probe design

The 19q13.11–q13.12, 27E7-28D3, 19q13.2–q13.31 and 19q13.32–q13.33 libraries were constructed from our public database of 32mer probe sequences^7^ (also see http://genetics.med.harvard.edu/oligopaints). The 89D–89E/BX-C and 89B–89D libraries consist of 42mer sequences discovered by OligoArray 2.1 (ref. 57) run with the following settings: -n 30 -l 42 -L 42 -D 1000 -t 85 -T 99 -s 70 -x 70 -p 35 -P 80 -m ‘GGGG;CCCC;TTTTT;AAAAA’ -g 44. The XIC Interstitial and Xist RNA libraries consist of 42mer sequences discovered by OligoArray 2.1 run with the following settings: -n 30 -l 42 -L 42 -D 1000 -t 75 -T 99 -s 70 -x 70 -p 35 -P 80 -m ‘GGGGGG;CCCCCC;TTTTTTT;AAAAAAA’ -g 44. The XIC HOPs and XIC 490 kb and 998 kb HOPs libraries were discovered using OligoArray 2.1 settings identical to those used for the XIC Interstitial and Xist RNA libraries, except ‘-n’ was set to 1. The 057/461 HOPs were discovered using OligoArray 2.1 settings identical to those used for the XIC HOPs except that ‘-t’ was set to 80. The 89B 5 kb and HoxB 5 kb libraries were discovered by OligoArray 2.1 run with the following settings: -n 30 -l 36 -L -D 1000 -t 80 -T 99 -s 75 -x 75 -p 35 -P 80 -m ‘GGGGGG;CCCCCC;TTTTTTT;AAAAAAA’ -g 38. Also see Supplementary Note 1.

### Construction of SV-40 T-antigen transformed MEF lines

To generate the CAST and 129 cell lines, primary MEFs were prepared from F1 embryos collected at embryonic day 13.5 from mice of either pure *M. musculus* (129S1/SvImJ) or *M. castaneus* (CAST/EiJ) backgrounds. MEFs were later immortalized by SV-40T antigen^58^ and subcloned by limiting dilution to obtain independent clones. The chromosome content of each subclone was screened by DNA FISH using probes against several autosomal genes.

### Cell culture

*Drosophila* clone 8 (DGRC 151) and S2R+ (DGRC 150) cells were obtained from the Drosophila Genomics Resource Center. S2R+ cells were grown in serum-supplemented (10%) Schneider’s S2 medium (serum SAFC 12103C; media Gibco 21720) at 25 °C. Clone 8 cells were grown in M3 medium (Sigma S3652) supplemented with serum (2%; SAFC 12103C), fly extract (2.5%) and 5 μg ml^−1^ insulin at 25 °C. WI-38 cells (ATCC CCL-75) cells were grown at 37 °C+5% CO_2_ in serum-supplemented (10%) DMEM (Dulbecco’s Modified Eagle Medium; serum Gibco 10437; media Gibco 10564). 129, CAST and EY.T4 129xCAST MEFs were grown in DMEM (Gibco 10313) supplemented with serum (15%, Gibco 10437) and GlutaMAX (Gibco 35050) at 37 °C+5% CO_2_. Penicillin and streptomycin (Gibco 15070) were also added to both insect and mammalian cell culture media to final concentrations of 50 U ml^−1^ and 50 μg ml^−1^, respectively.

### Preparation of sample slides for FISH

To prepare sample slides containing fixed insect and mammalian tissue culture cells for FISH, 100 μl of a 1 × 10^5^–1 × 10^6^ cells ml^−1^ suspension in rich media was spotted onto a poly-L-lysine coated slide and allowed to adhere for 1–3 h in tissue culture conditions (for example, 37 °C, 5% CO_2_ for mammalian cells). Slides were then washed in 1 × PBS, fixed in 1 × PBS+4% (w/v) paraformaldehyde for 10 min, rinsed in 1 × PBS, washed in 2 × saline-sodium citrate (SSCT), washed in 2 × SSCT+50% formamide (v/v) and finally transferred to 2 × SSCT+50% formamide for storage at 4 °C until use. For a stepwise protocol, please see the Oligopaints website ( http://genetics.med.harvard.edu/oligopaints); also see refs 7, 10). For STORM imaging, samples were prepared in the same way except that 22 × 30 mm #1.5 coverslips were used in place of microscope slides. For DNA-PAINT imaging, samples were prepared in the same way except that Lab-Tek II 8 chamber coverglass vessels (Nunc) were used in place of microscope slides and no poly-L-lysine was used.

### Two-colour co-localization FISH

FISH was performed with the 20–50 pmol of secondary probe simply being added to a 25 μl hybridization mix in parallel with 50 pmol of primary probe. Before hybridization, slides were warmed to RT, incubated for 2.5 min in 2 × SSCT+50% formamide at 92 °C, then incubated for 20 min in 2 × SSCT+50% formamide at 60 °C. A hybridization cocktail consisting of 2 × SSCT, 50% formamide, 10% (w/v) dextran sulfate, 10 mg of RNase (Fermentas EN0531) and Oligopaint probes was then added to the cells and sealed beneath a 22 × 22 mM #1.5 coverslip using rubber cement. Slides were denatured for 2.5 min at 92 °C on the top of a water-immersed heat block and allowed to hybridize overnight at 42 °C in a humidified chamber. The next day, the slides were washed for 15 min in 2 × SSCT at 60 °C, then for 10 min in 2 × SSCT at RT and then for 10 min in 0.2X SSC at RT. Slides were then mounted in SlowFade Gold+DAPI (Invitrogen S36938) under a 22 × 30 mM #1.5 coverslip and sealed with nail polish. For a stepwise FISH protocol, please see the Oligopaints website ( http://genetics.med.harvard.edu/oligopaints); also see refs 7, 10. In the instance where the secondary probe was added sequentially, the primary hybridization was performed as described above, except that the secondary probe was not included in the hybridization mix and the second and third wash steps were both shortened to 5 min. After these washes, 30 pmol of secondary probe was added in 25 μl of 2 × SSCT and sealed under a 22 × 30 mM #1.5 coverslip with rubber cement, then allowed to hybridize for the times indicated in Supplementary Fig. 7 at 60 °C on the top of a water-immersed heat block. The slides were then washed for 10 min in 2 × SSCT at 60 °C, then for 5 min in 2 × SSCT at RT, then for 5 min in 0.2 × SSC at RT and finally mounted as described above.

### 3D FISH for STORM

Sample coverslips were warmed to RT and then rinsed in 1 × PBT (1 × PBS+0.1% v/v Tween-20). Coverslips were then incubated in an aqueous 1 mg ml^−1^ NaBH_4_ solution for 7 min, then rinsed five times in 1 × PBT. Coverslips were then incubated in 1 × PBS+0.5% (v/v) Triton-X-100 for 10 min, then rinsed in 1 × PBT. Coverslips were then incubated for 30 min in 1 × PBS+20% (v/v) glycerol, and then flash-frozen by immersion into liquid nitrogen. Coverslips were allowed to thaw, placed back in 1 × PBS+30% glycerol, then flash-frozen again. This process was then repeated one additional time (three total flash-freezes). Coverslips were then rinsed in 1 × PBT, then incubated in 0.1N HCl for 5 min and then rinsed twice in 2 × SSCT. Coverslips were then incubated in 2 × SSCT+50% formamide (v/v) for 5 min and then incubated in 2 × SSCT+50% formamide at 60 °C for 20 min. At this point, 30 pmol of primary probe and 40 pmol of secondary probe were added to 25 μl of the hybridization cocktail described for ‘Two-colour co-localization FISH’ and the coverslips were sealed to glass slides using rubber cement (the glass slide acts as a ‘coverslip’ in this instance). Samples were denatured for 2.5 min at 78 °C on the top of a water-immersed heat block and allowed to hybridize overnight at 47 °C in a humidified chamber. The next day, the coverslips were washed as described for ‘Two-colour co-localization FISH’ and stored in 1 × PBS at 4 °C before mounting in STORM imaging buffer (see below) immediately before imaging. For a stepwise protocol, please see the Oligopaints website ( http://genetics.med.harvard.edu/oligopaints); also see reference 7.

### 3D FISH for DNA-PAINT imaging

FISH was performed as described for ‘3D (three-dimensional) FISH for STORM’ on transformed EY.T4 (ref. 52) fibroblasts, except that the 1 × PBS+glycerol and liquid nitrogen steps were omitted, and instead of being mounted in SlowFade Gold+DAPI samples were instead transferred to 1 × PBS supplemented with 500 mM NaCl and 5 nM ATTO 655-labelled 9-base imager strands^37^,^38^.

### XIC HOPs 3D FISH and simultaneous RNA/3D DNA FISH with HOPs

3D FISH was performed using a streamlined version of a previously reported simultaneous RNA FISH/3D DNA FISH protocol^7^. In brief, the slides were warmed to RT, rinsed in 1 × PBS and then rinsed in 1 × PBT. Slides were then incubated for 15 min in 1 × PBS+0.5% (v/v) Triton-X-100, then rinsed in 1 × PBT. Slides were then incubated for 5 min in 0.1N HCl and then rinsed three times in 2 × SSCT. Slides were then incubated in 2 × SSCT+50% formamide (v/v) for 5 min, then incubated in 2 × SSCT+50% formamide at 60 °C for 60 min. At this point, 40 pmol each of primary probe (129—AlexaFluor 488 label; CAST—ATTO 565 label; XIC Interstitial and Xist RNA—ATTO 633 label) and 50 pmol each of secondary probe (129–2X AlexaFluor 488-labelled Secondary 5; CAST—2X ATTO 565-labelled Secondary 1; XIC Interstitial and Xist RNA—2X ATTO 633-labelled Secondary 6) were added to 25 μl of the hybridization cocktail described for ‘Two-colour co-localization FISH.’ If RNA FISH was being performed, RNase was omitted from the hybridization cocktail. Slides were denatured for 3 min at 78 °C on the top of a water-immersed heat block and allowed to hybridize overnight at 47 °C. The next day, slides were washed and mounted as described for ‘Two-colour co-localization FISH.’ For a detailed protocol, please see the Oligopaints website ( http://genetics.med.harvard.edu/oligopaints).

### HOPs FISH on *Drosophila* salivary polytene chromosomes

A protocol from ref. 59 was used for the dissection and preparation of chromosome squashes from *Drosophila* salivary glands. FISH was then performed as described for ‘Two-colour co-localization FISH,’ with 20 pmol of primary Oligopaint probe set and secondary oligo being added per reaction for each probe used. Secondary oligos dual-labelled with AlexaFluor488, ATTO 565, and ATTO 633 were used with the 057 HOP, 461 HOP and BX-C probe set, respectively.

### Hybridization to whole-mount *Drosophila* ovarioles

A protocol modified from ref. 60) was used. Females of the genotype y^1#8^ (wild-type) were aged 24–48 h and then the ovaries were dissected in 1 × PBS. In brief, the dissected ovaries were fixed in a cacodylate fixative buffer^61^ for 4 min. During the fixation, the ovaries were teased apart towards the germarium tip. After the fixative was removed, the ovaries were transferred from the dissecting dish to a 0.5 ml Eppendorf tube and washed four times in 2 × SSCT. The ovaries were then gradually exchanged into 2 × SSCT+50% formamide (v/v) with a series of 10 minute washes in 2 × SSCT+20% formamide, then in 2 × SSCT+40% formamide and then two washes in 2 × SSCT+50% formamide. The ovaries were then predenatured in 2 × SSCT+50% formamide and heated to 37 °C for 4 h, 92 °C for 3 min and finally 60 °C for 20 min. Ovaries were then allowed to settle and the 2 × SSCT+50% formamide was removed before the addition of 36 μl of hybridization solution (2 × SSCT+50% formamide+10% (w/v) dextran sulfate) and 200 pmol each of primary Oligopaint probe sets suspended in a total volume ≤4 μl of ddH_2_O. The tissue and solution were gently mixed by flicking the tube and then heated to 91 °C in a thermal cycler for 3 min, followed by incubation overnight at 37 °C in the dark. Following the overnight incubation with primary probes, 2 × SSCT+50% formamide was added to the sample and washed for 30 min at 37 °C. Supernatant was removed and 200 pmol of each secondary oligo was then added in ∼50 μl of 2 × SSCT+50% formamide at 37 °C for 30 min. Following this incubation, two consecutive washes in 2 × SSCT+50% formamide were done at 37 °C, followed by one 10-min wash in 2 × SSCT+20% formamide and four rinses in 2 × SSCT, all at RT. After settling, excess 2 × SSCT was removed and the ovarioles were mounted in SlowFade Gold+DAPI (Invitrogen S36938).

### HOPs FISH in whole-mount *Drosophila* embryos

We collected embryos from overnight collections on apple juice plates. After collection, we dechorionated the embryos by submerging them in 50% bleach for 90 s, followed by a thorough wash in ddH_2_O. For fixation, embryos were placed in PBS containing 4% (w/v) formaldehyde, 0.5% (v/v) Nonidet P-40 and 50 mM EGTA, plus 500 μl Heptane for 30 min. The aqueous phase was removed and replaced with 500 μl MeOH and mixed vigorously for 2 min. The embryos were allowed to settle and were washed two times in 100% MeOH and stored for up to a week at −20 °C. Before FISH, the embryos were rehydrated in 2 × SSCT. FISH were then performed as described above for ovarioles.

### Wide-field and confocal microscopy and image processing

Slides were imaged using an Olympus IX-83 wide-field epifluorescent microscope using a 60X oil NA 1.42 lens and an Olympus XM-10 camera or a Zeiss LSM-780 laser scanning confocal microscope using a 63x oil NA 1.40 lens. Olympus images were captured and analysed using Olympus CellSens software, and Zeiss images were captured and analysed using Zeiss Zen software. Images were processed using the respective microscope-specific software and Adobe Photoshop.

### Quantification of FISH signals

FISH signals were counted manually using *Z*-stacks (that is, not using maximum *Z* projections). Two signals separated by an edge-to-edge distance of <1 μm were considered a single focus. The staining efficiency for a given channel (% labelling) indicates the number of nuclei with at least one focus in a given experiment. In two-colour experiments, % Co-localization indicates the percentage of signals produced by the secondary oligo that also had a co-localizing signal from the primary probe. Two signals were considered to be co-localized if their centre-to-centre distance was <250 nm for comparisons in *x* and *y* or <600 nm for comparisons using *Z*. These dimensions approximated an idealized diffraction-limited signal for the wavelengths of light used on our optical set-up. Measurements were adjusted to account for the chromatic aberration between the channels that was characterized using PSFj^62^ (please see Supplementary Fig. 5).

### Modelling of STORMm localizations on polymer structures

Polymers were simulated as follows. We first generated a random walk on a 3D lattice by adding monomers at random to open lattice points next to the growing end of a chain. Steps in each Cartesian direction were selected with equal probability, subject to the constraint that an accepted position be unoccupied by existing monomers. Growing chains that got stuck (more than 10 rejected moves) had their the terminal 10 monomers erased and were restarted growing. After assembling this initial random walk for the desired number of monomers, we used the Bond Fluctuation Method^63^ and Pivot Algorithm^64^,^65^ to equilibrate the polymer. Polymer chains were converted to STORM images by assigning to each monomer a random number of switching cycles, drawn from an exponential distribution as observed for switching of Cy5 (ref. 66). A small number of background localizations with uniform spatial distribution were then added to the position list. Gaussian white noise was added to the position of each localizations to account for limited localization precision. These final ‘dye’ positions were rendered as STORM images in an identical fashion to that used for our raw dye localization data following spot fitting. To simulate the effect of reduced localizations, a random subset of the total localizations was removed before rendering. Parameters used: Number of monomers=600 or 1,500, mean number of localizations =2, sigma for localization precision localization=1 monomer diameter.

### STORM microscopy

STORM images were taken on a customized Olympus IX-71 inverted microscope configured for high angle oblique incidence excitation with a 647nm laser and × 100 1.43 NA oil-immersion objective. Microscope construction was previously described^66^. STORM imagimg was performed in TN buffer (50 mM Tris (pH 8.0) and 10 mM NaCl) containing an oxygen scavenging system composed of 0.5 mg ml^−1^ glucose oxidase (Sigma-Aldrich), 40 μg ml^−1^ catalase (Roche or Sigma-Aldrich) and 10% (w/v) glucose), using 1% (v/v) 2-mercaptoethanol as a thiol. Also see ref. 66. Samples were selected in an experimenter-blind manner and imaged at 60 Hz for 32,000–65,000 frames (based on molecule localization rate). Photoactivation of dyes was tuned with a 405 laser for which the intensity was increased slowly throughout the image acquisition from 0 mW towards a maximum intensity of 1 mW to maintain an approximately uniform molecule localization rate for the first half of the acquisition. The same rate of 405 amplification was used for all cells imaged within a sample.

### STORM image construction

Molecule localization movies were fit using the 3D-DAOSTORM algorithm^67^. Localizations were plotted as single points or as Gaussian spots with widths normalized to the number of photons measured per localization using custom software written in MATLAB (see https://github.com/ZhuangLab/matlab-storm). The average photons per localization was >4,000. STORM images were constructed from the registration of Cy5 single-molecule fluorescence events, and no appreciable foci were detected in the absence of primary Oligopaint probe (data not shown). Single-molecule fluorescence events were localized with an average precision of ∼9 nm (s.d.) and a resolution (FWHM) of ∼20 nm.

### DNA-PAINT microscopy

Fluorescence imaging was carried out on an inverted Nikon Eclipse Ti microscope (Nikon Instruments) with the Perfect Focus System, applying an objective-type TIRF configuration using a Nikon TIRF illuminator with an oil-immersion objective (CFI Apo TIRF × 100, NA 1.49, Oil) yielding a pixel size of 160 nm. Two lasers were used for excitation—488 nm (200 mW nominal, Coherent Sapphire) and 647 nm (300 mW nominal, MBP Communications). The laser beam was passed through clean-up filters (ZT488/10 and ZET640/20, Chroma Technology) and coupled into the microscope objective using a multi-band beam splitter (ZT488rdc/ZT561rdc/ZT640rdc, Chroma Technology). Fluorescence light was spectrally filtered with emission filters (ET525/50 m and ET700/75 m, Chroma Technology) and imaged on an EMCCD camera (iXon X3 DU-897, Andor Technologies). Images were acquired with a CCD readout bandwidth of 3 MHz at 14 bit, 5.1 pre-amp gain and no electron-multiplying gain using the centre 256 × 256 px of the CCD chip. Imaging was performed using highly inclined and laminated optical sheet illumination^39^ with an excitation intensity of ∼50 mW using the 647 nm laser line. A total of 15,000 frames at a frame rate of 10 Hz were collected, resulting in ∼25 min imaging time.

### DNA-PAINT image construction

Super-resolution DNA-PAINT images were reconstructed using spot-finding and two-dimensional Gaussian fitting algorithms implemented in LabVIEW^37^,^38^. Localizations are represented Gaussian spots with widths normalized to the localization accuracy. All DNA-PAINT images were constructed from ATTO 655 localizations and co-localized with a diffraction-limited ATTO 488 focus. A simplified version of the DNA-PAINT software is available for download at http://www.dna-paint.net/ or http://molecular-systems.net/software/. Single-molecule fluorescence events were localized with an average precision of 6.5 nm (s.d.) and a resolution (FWHM) of ∼15.3 nm.

## Additional information

**How to cite this article**: Beliveau, B.J. *et al.* Single-molecule super-resolution imaging of chromosomes and in situ haplotype visualization using Oligopaint FISH probes. *Nat. Commun.* 6:7147 doi: 10.1038/ncomms8147 (2015).

## Supplementary Material

Supplementary Information Supplementary Figures 1-20, Supplementary Tables 1-4 and Supplementary Note 1
