# Supplementary material for: Single-molecule super-resolution imaging of chromosomes and in situ haplotype visualization using Oligopaint FISH probes
Source: Nat Commun. 2015 May 12;6:7147. doi: 10.1038/ncomms8147 (PMC4430122; doi:10.1038/ncomms8147)
Supplement: Supplementary Information — Supplementary Figures 1-20, Supplementary Tables 1-4 and Supplementary Note 1 [file ncomms8147-s1.pdf]

Supplementary. Figure 1

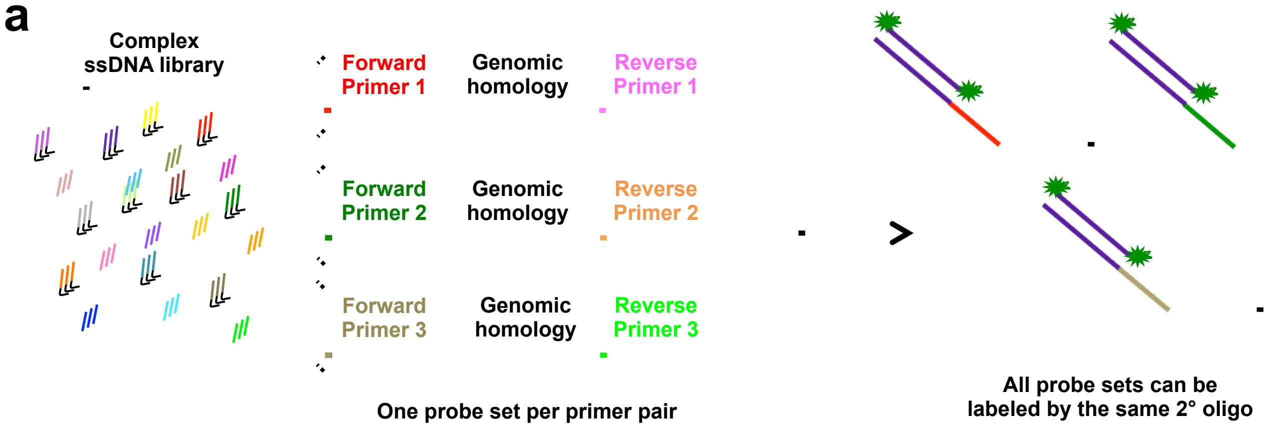

Supplementary Figure 1 The use of secondary (2°) oligos facilitates multiplexing. A multiplexed Oligopaints library contains multiple probe sets, each carrying its own unique primer sequences. The use of labeled secondary oligos allows every probe set in a multiplexed library to be visualized with the same oligo.

S. Figure 2

a

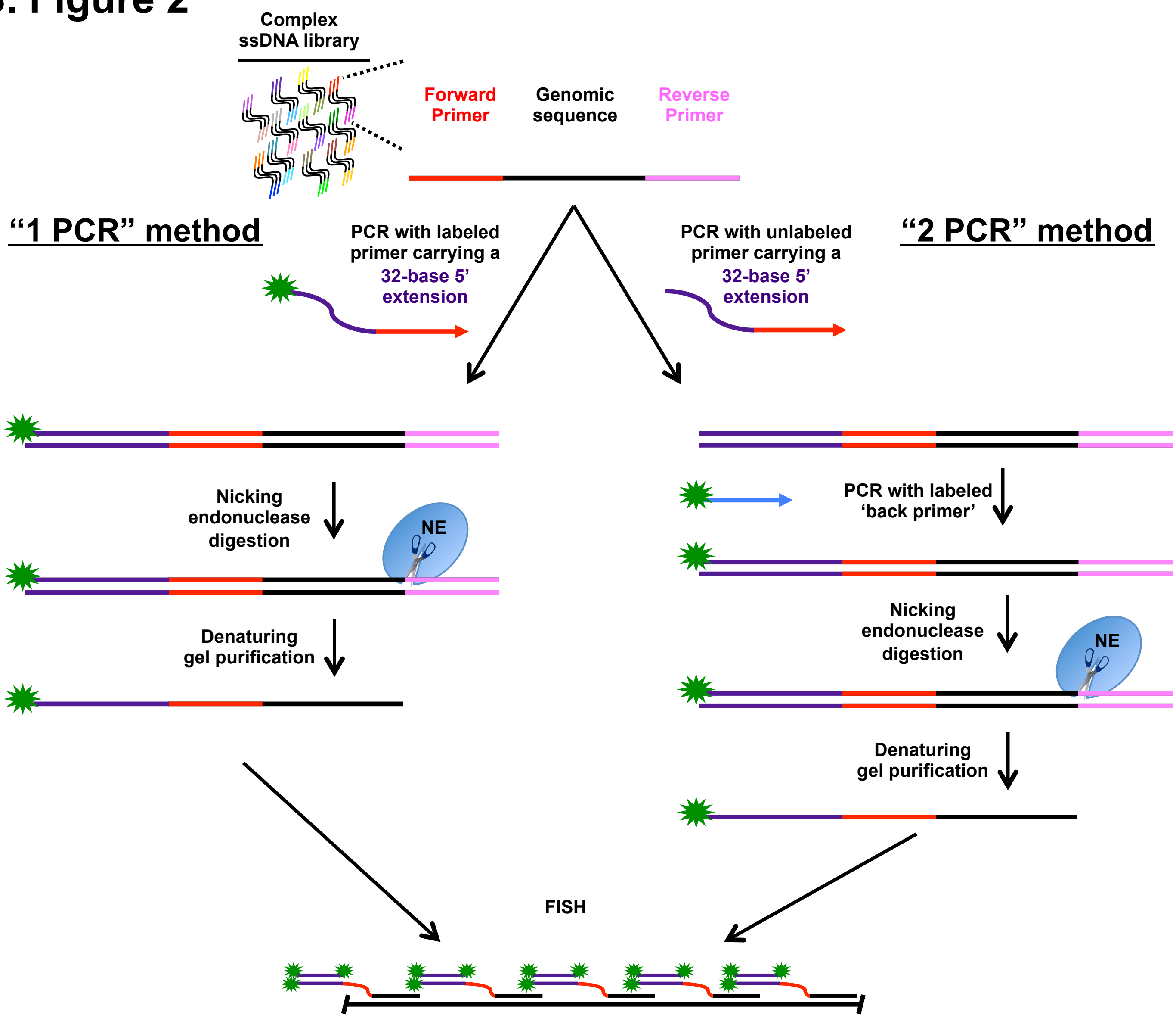

b

2° oligo	Back Primer Sequence 5' -> 3'
1	CACCGACGTCGCATAGAACGG
2	CGCAGCTCCACTTGATCTCGC
3	CGAGCCAGGTCATCCTAGCC
4	GGTGTGGCTCGGTATCGTGC
5	TAGCGCAGGAGGTCCACGAC
6	CACACGCTCTCCGTCTTGGC

Supplementary Figure 2 Two strategies used to introduce a 5' label and a secondary (2°) oligo binding sequence into Oligopaint probe sets. (**a**) Cartoons illustrating the two strategies. (**b**) A list of experimentally tested primer sequences for use in the "2 PCR" method.

S. Figure 3

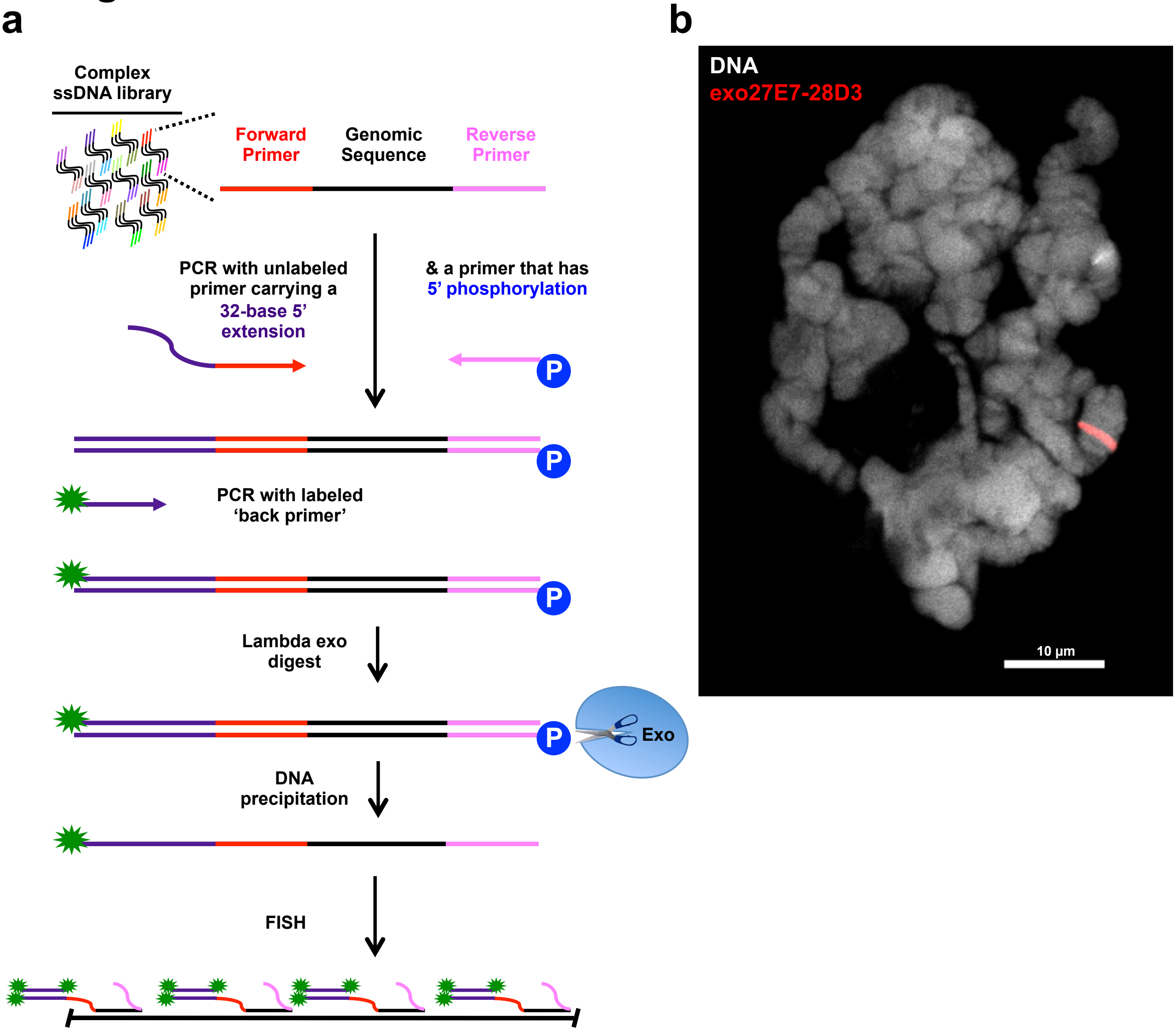

Supplementary Figure 3 A one-day Oligopaints synthesis strategy (**a**) Oligopaints can also be synthesized using a method that renders the products of PCR single-stranded via lambda exonuclease. Here, we show how this protocol can be used after a 32-base 5' extension has been added to Oligopaint oligos via touch-up PCR. Note that the use of lambda exonuclease leaves a MainStreet at both ends of the oligo probe. (**b**) Representative image of FISH performed using a probe produced via the lambda exonuclease method. 30 pmol of a Cy3-labeled Oligopaint probe set targeting the 27E7-28D3 region of the left arm of Drosophila chromosome 2 (red) was hybridized to spread salivary gland polytene chromosomes (**Methods**). DNA is stained with DAPI (gray). Image is a single Z slice. Note: We have also used probes produced using lambda exonuclease on cells from Drosophila, mouse, and human cell culture as well as Drosophila tissues.

Supplementary. Figure 4

a

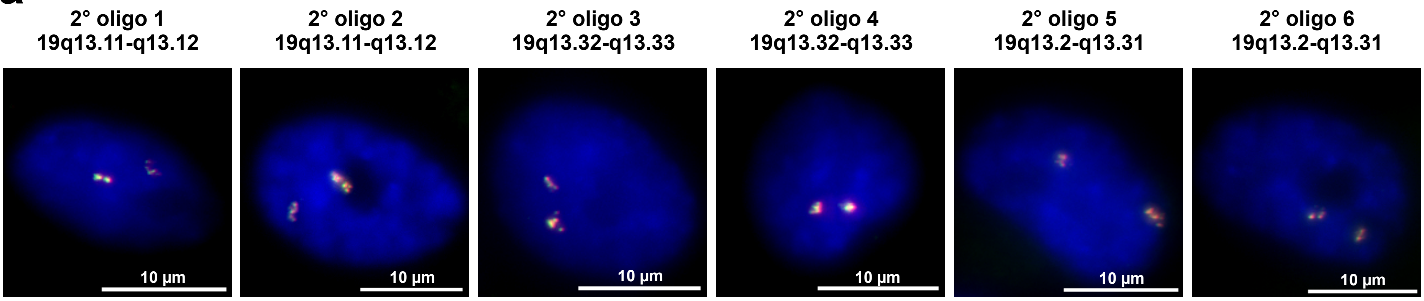

b

				Staining Pattern							
**2° oligo**	**Target**	**Span**	***n***	**0 1°, 0 2°**	**1 1°, 1 2°**	**2 1°, 2 2°**	**3 1°, 3 2°**	**4 1°, 4 2°**	**5 1°, 5 2°**	**% Labeling**	**% Co-localization**
1	4p16.1	52 kb	112	3.6%	5.4%	87.5%	3.6%	00.0%	00.0%	96.4	100
1	19q13.11-q13.12	3.0 Mb	111	00.0%	10.8%	74.8%	13.5%	0.9%	0%	100	100
2	19q13.11-q13.12	3.0 Mb	121	00.0%	14.9%	71.1%	11.6%	2.5%	0%	100	100
3	19q13.32-q13.33	2.3 Mb	126	00.0%	14.3%	69.8%	12.7%	3.2%	0%	100	100
4	19q13.32-q13.33	2.3 Mb	112	00.0%	8.9%	82.1%	8.9%	0%	0%	100	100
5	19q13.2-q13.31	2.1 Mb	120	00.0%	13.3%	75.0%	11.7%	0%	0%	100	100
6	19q13.2-q13.31	2.1 Mb	144	00.0%	12.5%	77.1%	6.9%	2.8%	0.7%	100	100

Supplementary Figure 4 Co-localization of primary (1°, Oligopaint) probe sets and secondary (2°) oligos in diploid human WI-38 cells. Oligopaint probe (red) and secondary oligos (green) were added simultaneously and allowed to hybridize. (**a**) Images for each secondary oligo, which were captured on a wide-field epifluorescent microscope and are maximum Z projections. For an image of secondary oligo 1 at 4p16.1, please see **Fig. 1c**. Note that a chromatic aberration on our wide-field microscope results in the green channel appearing slightly to the left of the red channel. For more details, please see **Supplementary Fig. 5**. (**b**) Staining patterns observed. *n* = nuclei scored, see **Methods** for % Labeling and % Co-localization. Similar results were observed in Drosophila clone 8 cells (data not shown).

Supplementary. Figure 5

a

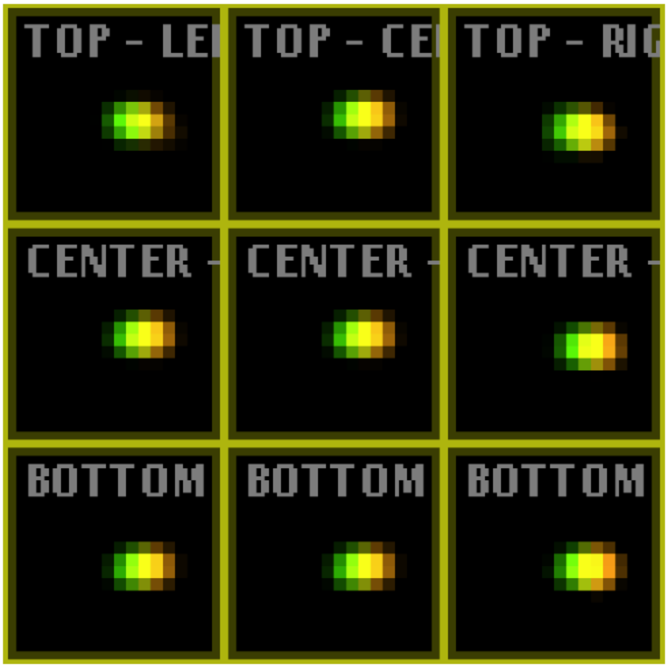

Average X shift: +177.2 nm

Average Y shift: -5.1 nm

Average Z shift: -55.5 nm

***n* = 34 beads**

b

PSFj Output
250 nm TetraSpeck™ fluorescent beads

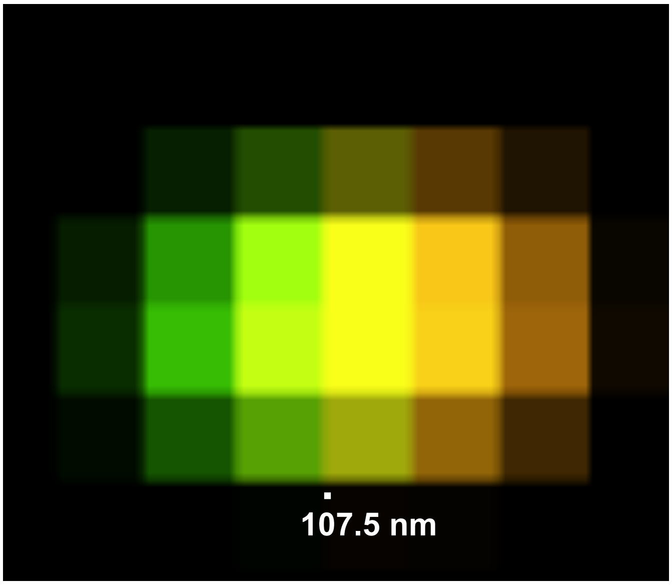

FISH
316 kb 89D-89E 1° + 2° oligo 1

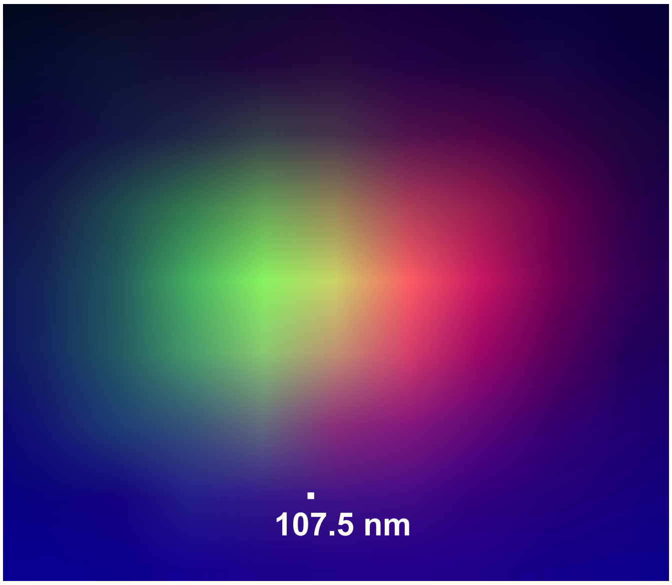

Supplementary Figure 5 Characterizing the chromatic aberration on our wide-field epifluorescent microscope. (**a**) PSFj (reference 62) was run on 250 μm TetraSpeck™ (Invitrogen) beads – which are directly conjugated with blue, green, red, and far-red dyes – to compare the red and green channels. Left: An "average bead" montage, in which the field of view has been divided into 9 parts. For each, the signals of all beads in that part are averaged and displayed as a two-color image of an "average" bead. Note that our chromatic aberration occurs consistently in all parts. Right: Summary data for all 34 beads imaged. (**b**) A comparison of the PSFj output from the TetraSpeck™ beads and two-color co-localization FISH. Left: a zoomed-in view of the Center-Center average bead. Right: A zoomed-in view of a FISH focus from a two-color co-localization experiment performed in Drosophila clone 8 cells.

Supplementary Figure 6

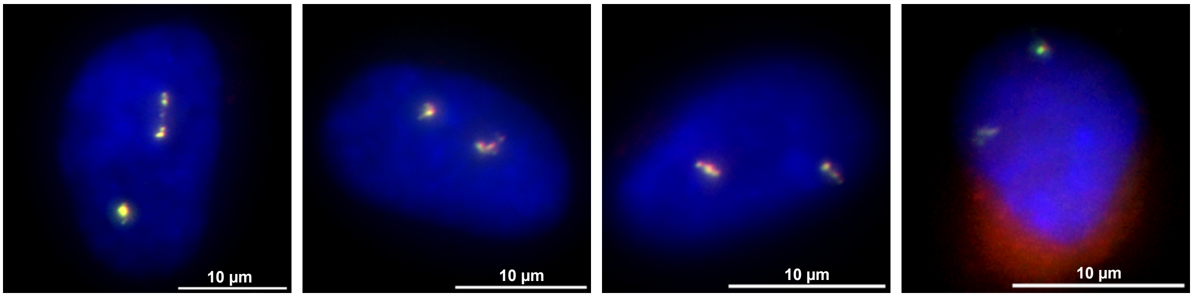

2° hyb. duration:	5 minutes	15 minutes	30 minutes	60 minutes
1° signal:	++	+++	+++	+
1° background:	++++	+++	+	+++
2° signal:	++++	++++	++++	++
2° background:	+	+	+	+
n:	53	51	55	55
% Labeling 1°:	100	100	100	100
% Labeling 2°:	100	100	100	100
% Co-localization:	98.2	95.1	99.1	99.1

Supplementary Figure 6 Sequential addition of labeled secondary (2°) oligos in diploid human WI-38 cells. 30 pmol of Cy3 labeled primary (1°, Oligopaint) probe set (red) consisting of 20,020 oligos targeting 3.0 megabases at 19q13.11-q13.12 was added and allowed to hybridize overnight at 42°C. Slides were then washed, incubated at 60°C with 30 pmol of 2X Alexa647 2° oligo 1 (green) in 2X SSCT for the indicated amount of time, washed, and mounted (**Methods**). Images and qualitative assessments of the signal and background of the primary and secondary probes on a scale from "-" (undetectable) to "+++++" are given for each experiment. *n* = nuclei scored, see **Methods** for % Labeling and % Co-localization. Images were captured on a wide-field epifluorescent microscope and are maximum Z projections. Note that a chromatic aberration on our wide-field microscope results in the green channel appearing slightly to the left of the red channel. For more details, please see **Supplementary Fig. 5**.

Supplementary. Figure 7

a

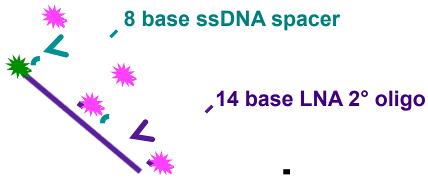

b

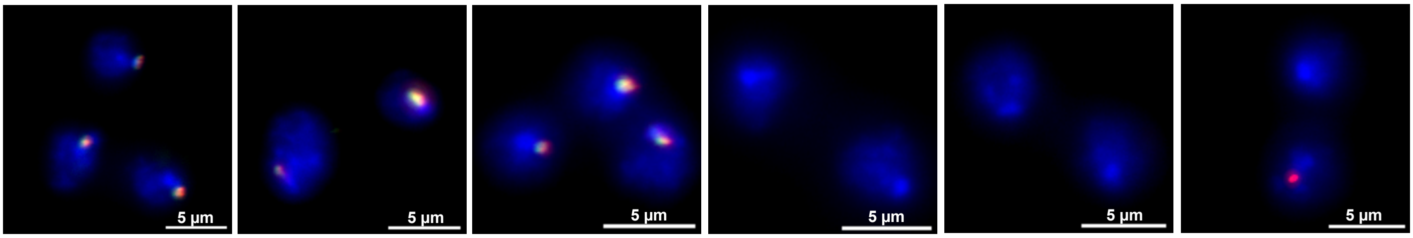

c

Staining Pattern

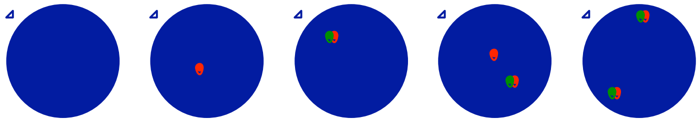

2° oligo	Target	*n*	0 1°, 0 2°	0 1°, 1 2°	1 1°, 1 2°	1 1°, 2 2°	2 1°, 2 2°	% labeling 1°	% labeling 2°	% Co-local -ization
LNA1	359	136	1.5%	0%	97.8%	0%	0.7%	98.5	98.5	100
LNA2	359	116	0%	0%	96.6%	0.9%	2.6%	100	100	100
LNA3	359	126	0%	0%	100%	0%	0%	100	100	100
LNA1	N/A	139	100%	0%	0%	0%	0%	0	0	N/A
LNA2	N/A	134	100%	0%	0%	0%	0%	0	0	N/A
LNA3	N/A	176	97.7%	2.3%	0%	0%	0%	0	2.3	N/A

Supplementary Figure 7 Locked nucleic acids (LNAs) as secondary (2°) oligos. (**a**) The design used to test LNAs at the 359 satellite sequences on the Drosophila X chromosome. (**b**) Images of FISH performed in diploid Drosophila clone 8 cells. 40 pmol of 2X TYE563 labeled LNA secondary oligo (red) was added in the presence or absence of 20 pmol of a 6-FAM labeled primary oligo (green) and allowed to hybridize overnight at 42°C. Images were captured on a wide-field epifluorescent microscope and are maximum Z projections. Note that a chromatic aberration on our wide-field microscope results in the green channel appearing slightly to the left of the red channel. For more details, please see **Supplementary Fig. 5**. (**c**) Staining patterns observed. *n* = nuclei scored, see **Methods** for % Labeling, % Co-localization.

Supplementary. Figure 8

a

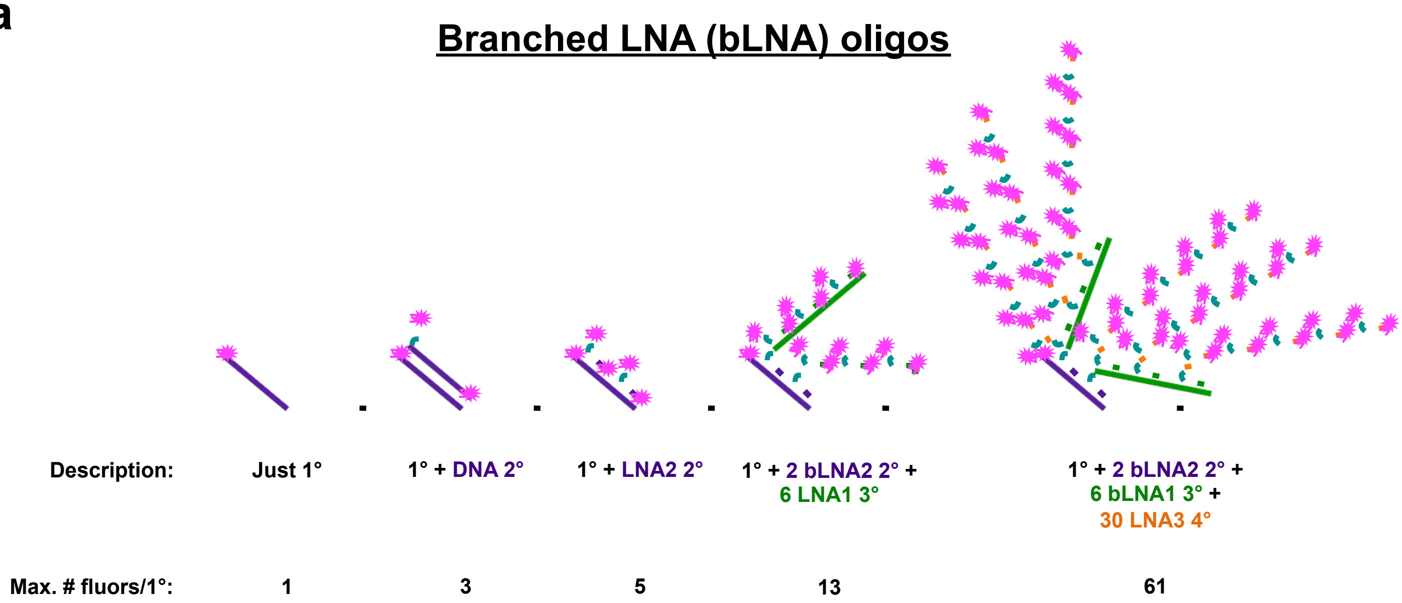

b

Description	Sequence 5' -> 3'
359 1° with 2° oligo 1 binding site	/5TYE563/CACCGACGTCGCATAGAACGGAAGAGCGTGTGTTTTTTTTTTAGGGATCGTTAGCACTGGTAATTAGCTGC
2° oligo 1	/5TYE563/CACACGCTCTTCCGTTCTATGCGACGTCGGTGTTTTTTTT/3TYE563/
359 1° with 2 LNA2 binding sites	/5TYE563/TTTGCTCGACCTGGTCTTTGCTCGACCTGGTCTTTTTTTTTTAGGGATCGTTAGCACTGGTAATTAGCTGC
Labeled LNA2	/5TYE563/G+AC+CA+GG+TC+GA+GC+ATTTTTTTT/3TYE563/
LNA2 with 3 LNA1 binding sites	G+AC+CA+GG+TC+GA+GC+ATTTTTTTTTTGCACGTCGTGGATTTTGCACGTCGTGGATTTTGCACGTCGTGGA
Labeled LNA1	/5TYE563/T+CC+AC+GA+CG+TG+CA+ATTTTTTTT/3TYE563/
LNA1 5 LNA3 binding sites	T+CC+AC+GA+CG+TG+CA+ATTTTTTTTACCGGTGCTAGTCGTTACCGGTGCTAGTCGTTACCGGTGCTAGTCGTTACCGGTGCTAGTCGTTACCGGTGCTAGTCG
Labeled LNA3	/5TYE563/C+GA+CT+AG+CA+CC+GG+TTTTTTTTT/3TYE563/

Supplementary Figure 8 Branched LNA (bLNA). (**a**) Cartoons illustrating the binding of secondary (2°), tertiary (3°), and quaternary (4°) oligos to a primary (1°) oligo probe. For each, the number of fluorophores associated the primary oligo if all binding sites were occupied is given. (**b**) Sequences of oligos used in bLNA experiments. A "+" sign preceding a base indicates an LNA residue. The Integrated DNA Technologies modification codes '/5TYE563/' and '/3TYE563/' indicate 5' and 3' TYE563 molecules.

Supplementary. Figure 9

a

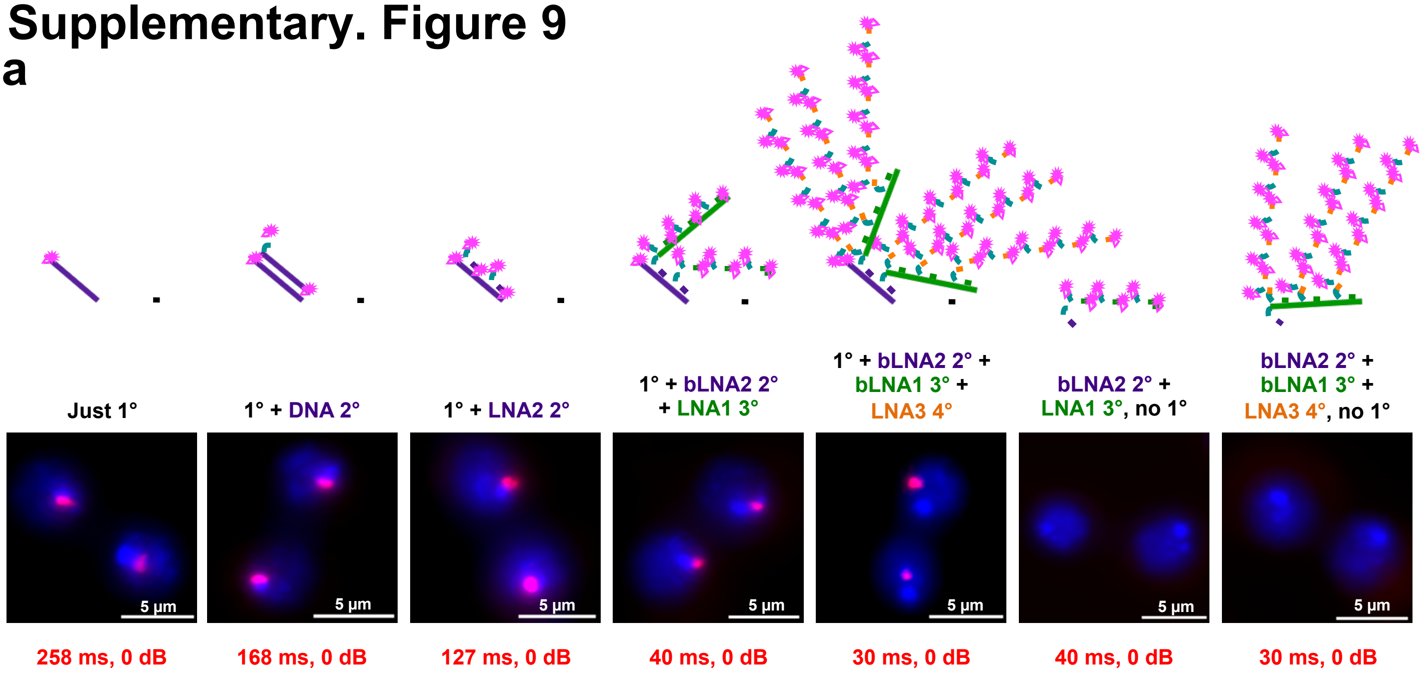

b

Staining Pattern

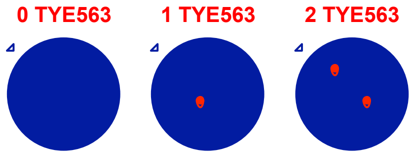

Oligos added	*n*	0 TYE563	1 TYE563	2 TYE563	% Labeling
Just 1°	147	0%	100%	0%	100
1° + DNA 2°	167	0.6%	98.8%	0.6%	99.4
1° + LNA2 2°	173	0%	97.7%	2.3%	100
1° + bLNA2 2° + LNA1 3°	144	0%	100%	0%	100
1° + bLNA2 2° + bLNA1 3° + bLNA3 4°	160	0.6%	98.1%	1.3%	99.4
bLNA2 2° + LNA1 3°, no 1°	118	99.2%	0.8%	0%	0.8
bLNA2 2° + bLNA1 3° + bLNA3 4°, no 1°	149	99.3%	0.7%	0%	0.7

Supplementary Figure 9 Branched LNA (bLNA) at the Drosophila 359 satellite sequences. (**a**) FISH with LNA secondary (2°) oligos in diploid Drosophila clone 8 cells. 20 pmol of TYE563 labeled primary (1°) probe oligo targeting the 359 satellite on chromosome X (red) was added in the presence or absence of the indicated secondary, tertiary (3°), and quaternary (4°) oligos; 20 pmol of each bLNA oligo and 40 pmol of each 2X TYE563 labeled LNA oligo were added. Hybridizations were performed overnight at 42°C. Images from each are shown, below which are the exposure settings used to acquire TYE563. Images were captured on a wide-field epifluorescent microscope and are maximum Z projections. (**b**) Staining patterns observed. *n* = nuclei scored, see **Methods** for % Labeling.

Supplementary. Figure 10

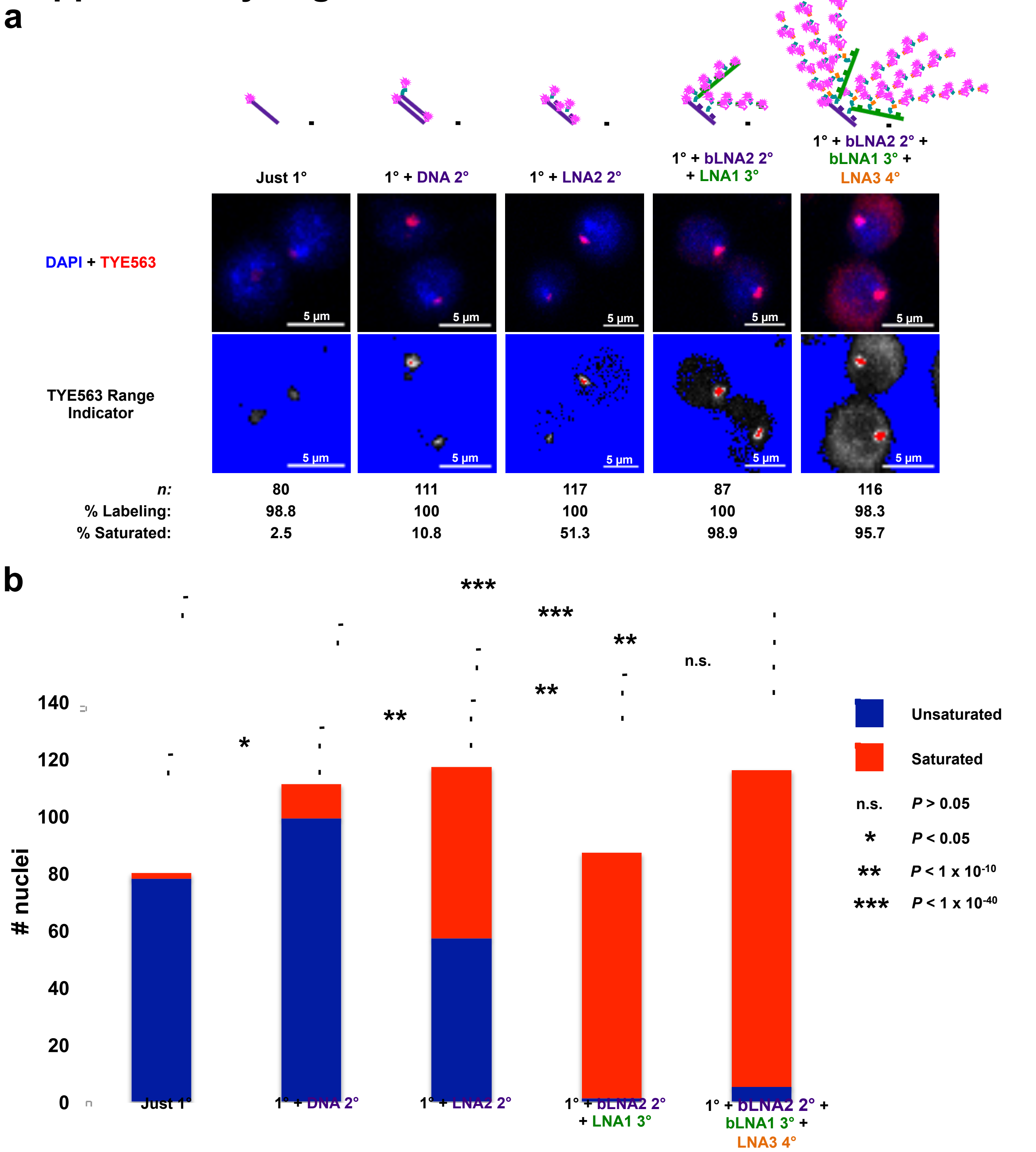

Supplementary Figure 10 Branched LNA (bLNA) leads to stronger signals. (**a**) The indicated samples (described in **Supplementary Fig. 9**) were imaged on a Zeiss LSM-780 laser scanning confocal microscope with a constant gain setting (master gain 700, digital gain 1.5). Representative images are shown as pseudocolored micrographs and with the 'Range Indicator' look up table for TYE563. Red pixels indicate saturation. *n* = the number of nuclei, % Labeling = percent nuclei with at least one signal, % Saturated = percent nuclei with at least one saturated focus. (**b**) The distribution of signals for each experiment. *P* values were calculated using a two-tailed Fisher's exact tests. 'n.s.' = not significant.

Supplementary. Figure 11

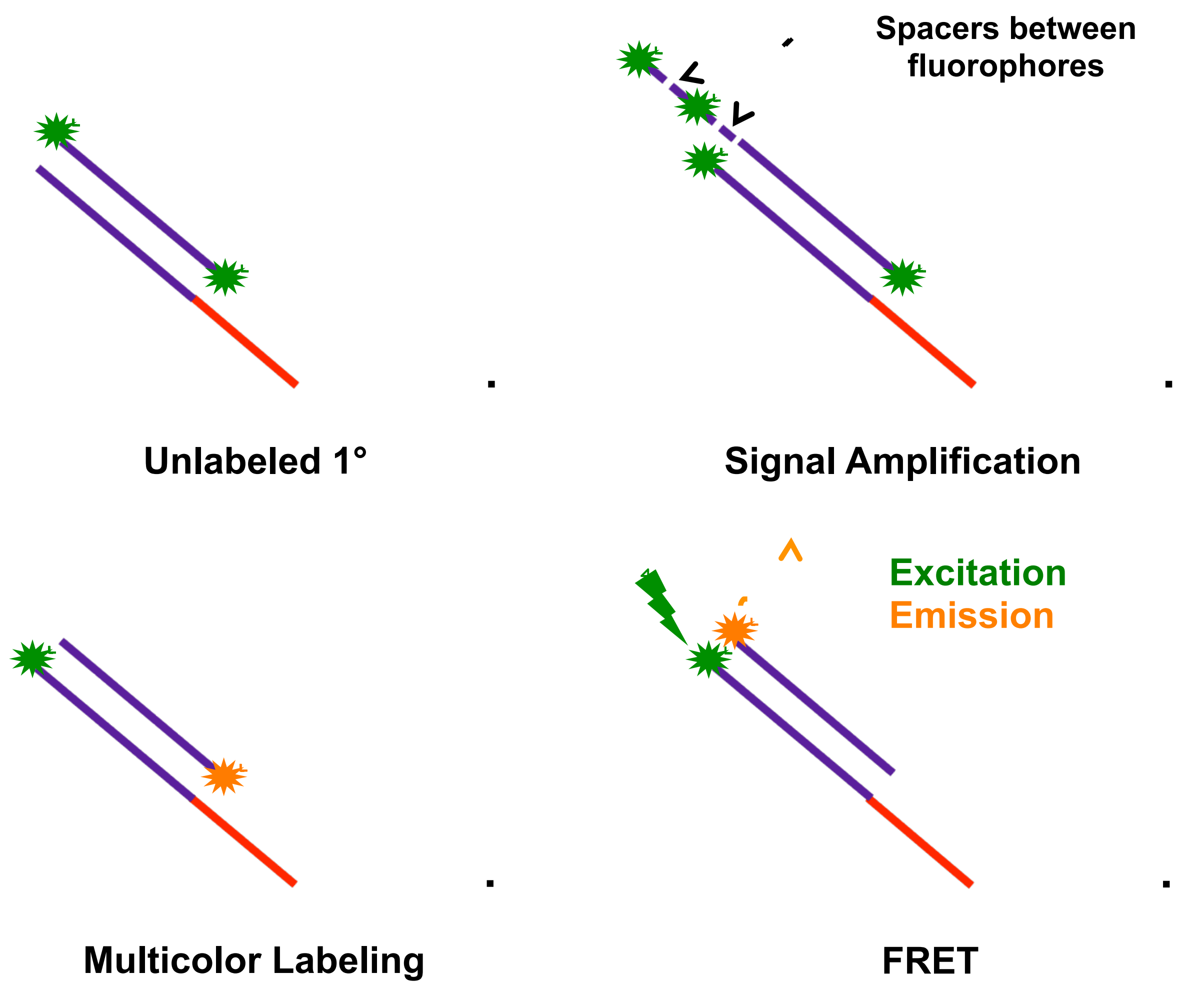

Supplementary Figure 11 Multiple applications enabled by MainStreet and secondary (2°) oligos. In addition to facilitating multiplexing, secondary oligos could amplify signal by carrying multiple copies of the dye present on the primary (1°) probe. Primary-secondary dye pairings can also be engineered for applications such as multicolor labeling and Förster resonance energy transfer (FRET).

Supplementary. Figure 12

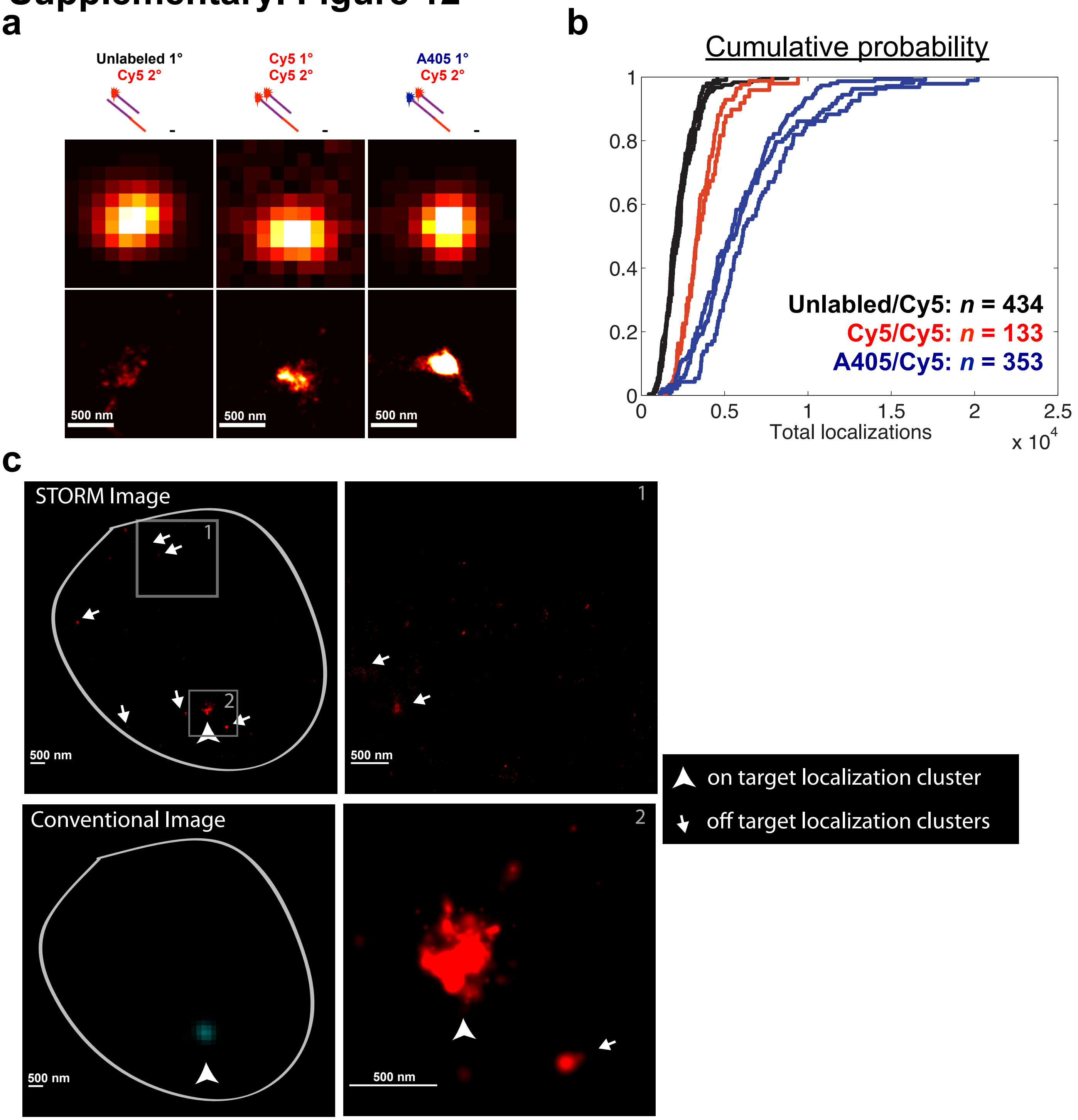

Supplementary Figure 12 Oligopaints primary-secondary pairings and STORM. (**a**) Images of the Drosophila BX-C produced using the three indicated primary-secondary pairings. Top row: conventional, diffraction-limited images. Bottom row: STORM images of the same foci. (**b**) Cumulative distribution plot of the number of single-molecule localizations observed per BX-C focus for each of the three primary-secondary pairings. Separate curves in the same color indicate biological replicates, which were combined to produce the medians plotted in **Fig. 2c**. *n* = the number of foci imaged per replicate. (**c**) Examples of off-target background staining. A single nucleus is shown, with the perimeter approximately outlined. The arrowhead indicates the “on target” cluster of localizations corresponding to the BX-C locus that clearly overlap with the signal in the conventional image. Small independent clusters of localizations are often observed elsewhere in the nucleus (arrows), in addition to stray localizations.

Supplementary. Figure 13

a

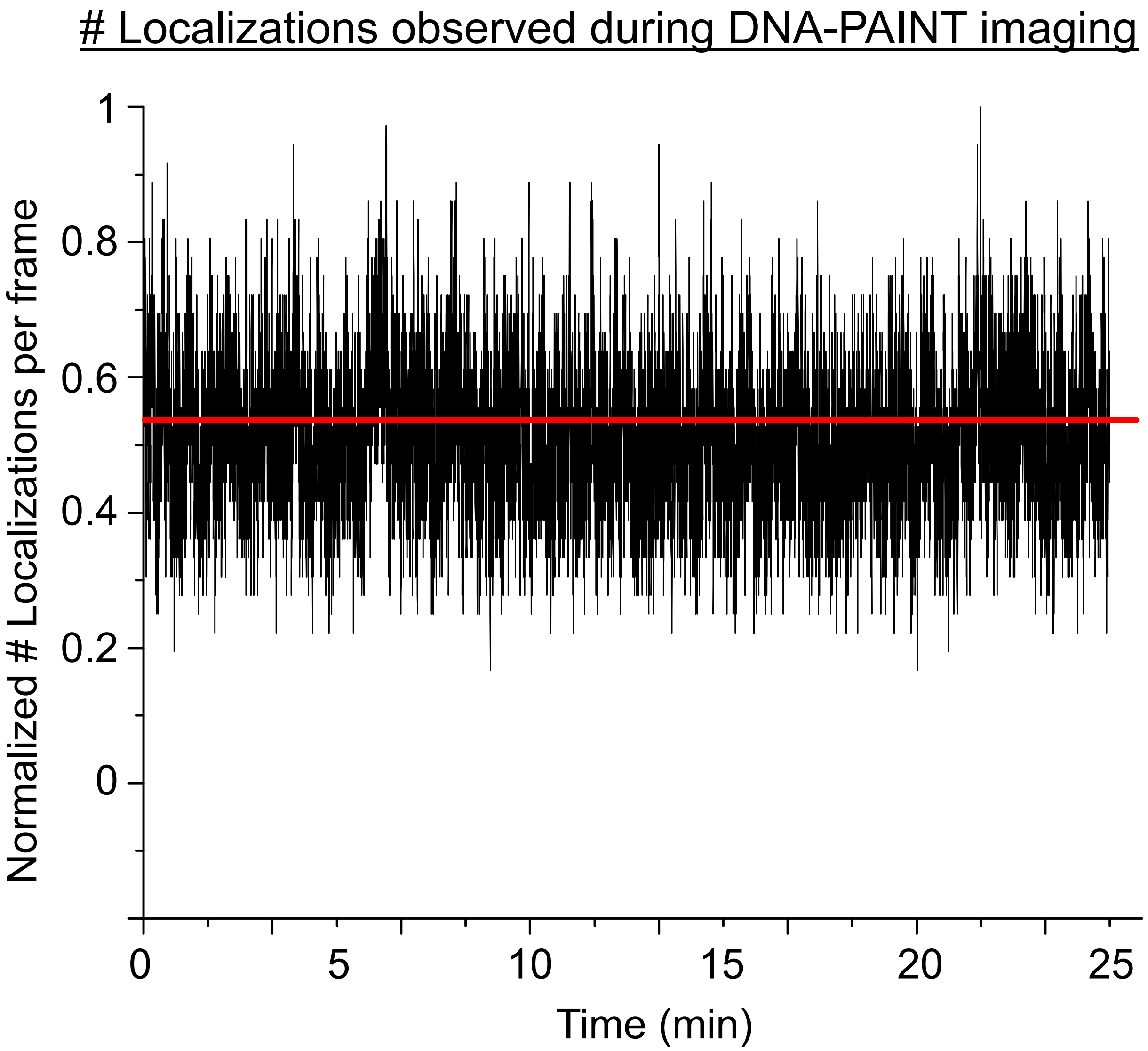

Supplementary Figure 13 The number of single molecule localization events (y-axis) remains relatively constant over time (x-axis) during DNA-PAINT imaging, demonstrating that photo-bleaching does not noticeably reduce the localization rate over the time-scale used for imaging. Localization number is normalized relative to the maximum # of localizations observed in a single frame for presentation. A line with a slope of 0 and an Y-intercept equal to the mean # of normalized localizations per frame (0.54) is provided for comparison. Data is taken from the field imaged in **Fig. 3c**, top row.

S. Figure 14

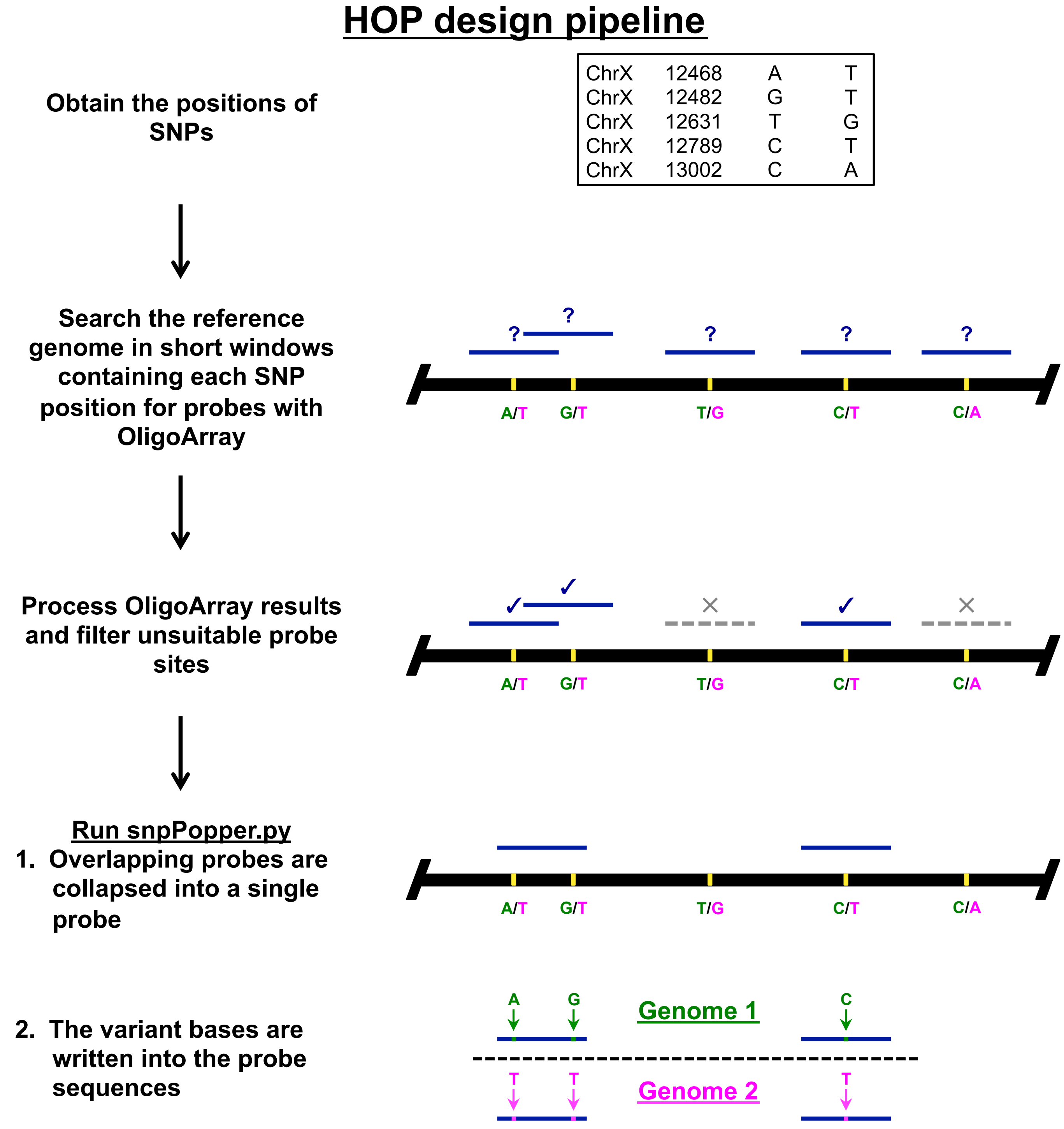

Supplementary Figure 14 HOP design pipeline using OligoArray (reference 57) and snpPopper.py.

Supplementary. Figure 15

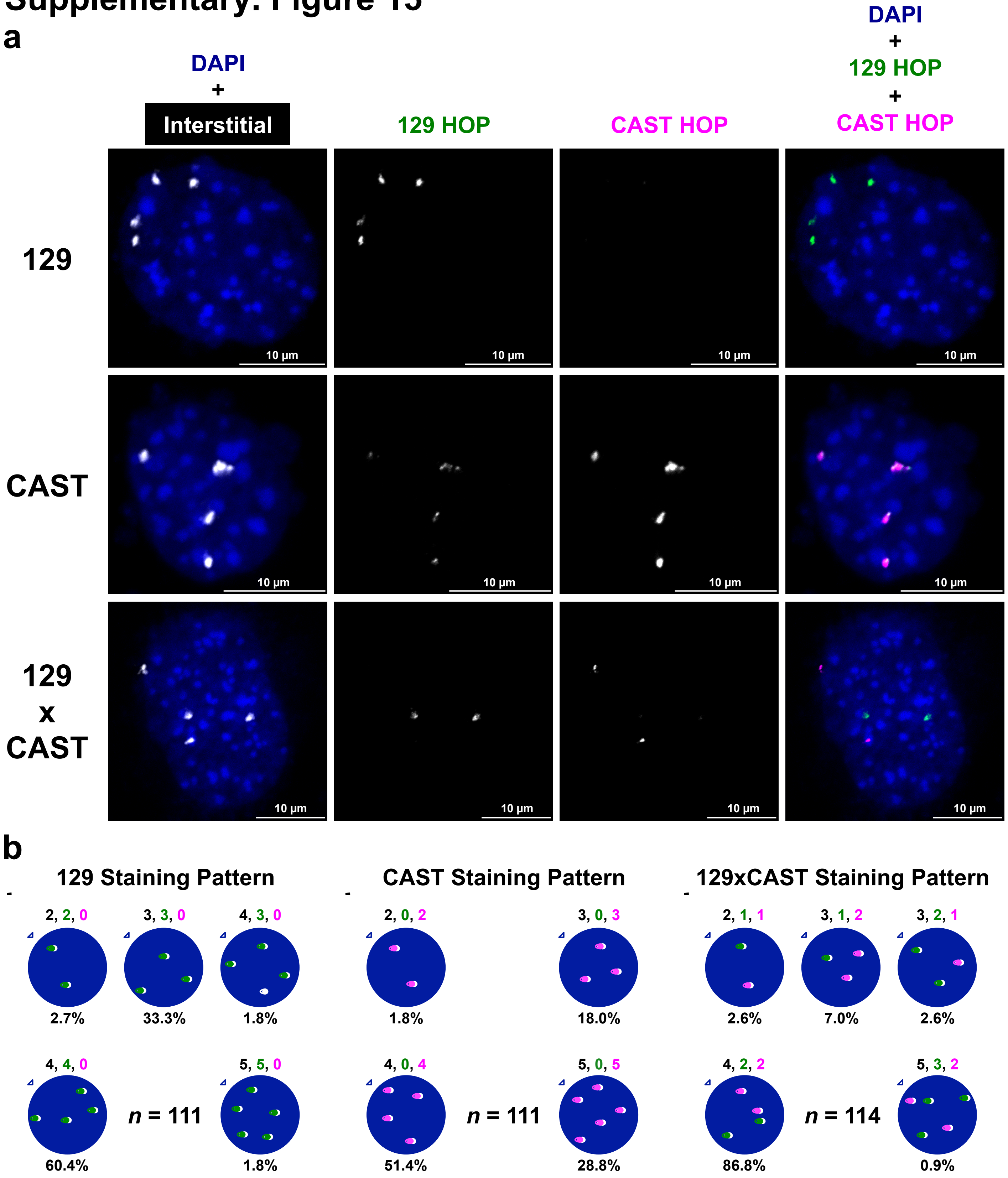

Supplementary Figure 15 HOPs targeting the mouse X-inactivation center. (**a**) FISH performed in the three indicated MEF lines. Images were captured on a laser scanning confocal microscope and are maximum Z projections. (**b**) Staining patterns observed. *n* = nuclei scored.

Supplementary. Figure 16

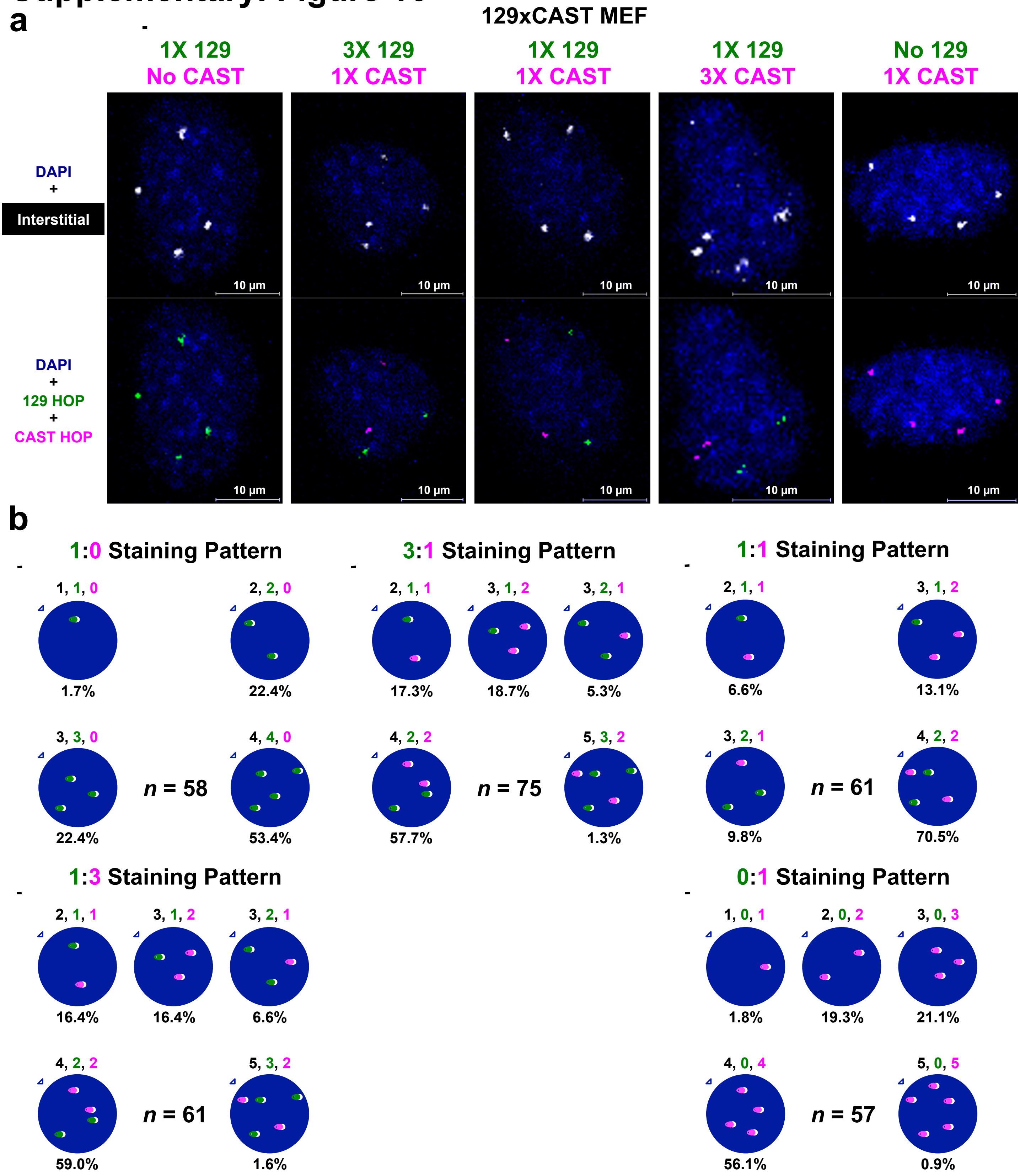

Supplementary Figure 16 Concentration-dependent effects on HOP staining patterns. (**a**) FISH performed in the hybrid EY.T4 129xCAST line. Each sample received the XIC 'Interstitial' probe set (white) and the indicated amount of the 129 (green) and CAST (magenta) XIC HOPs. DNA is stained with DAPI (blue). Images were captured on a laser scanning confocal microscope and are maximum Z projections. (**b**) Staining patterns observed. *n* = nuclei scored.

Supplementary. Figure 17

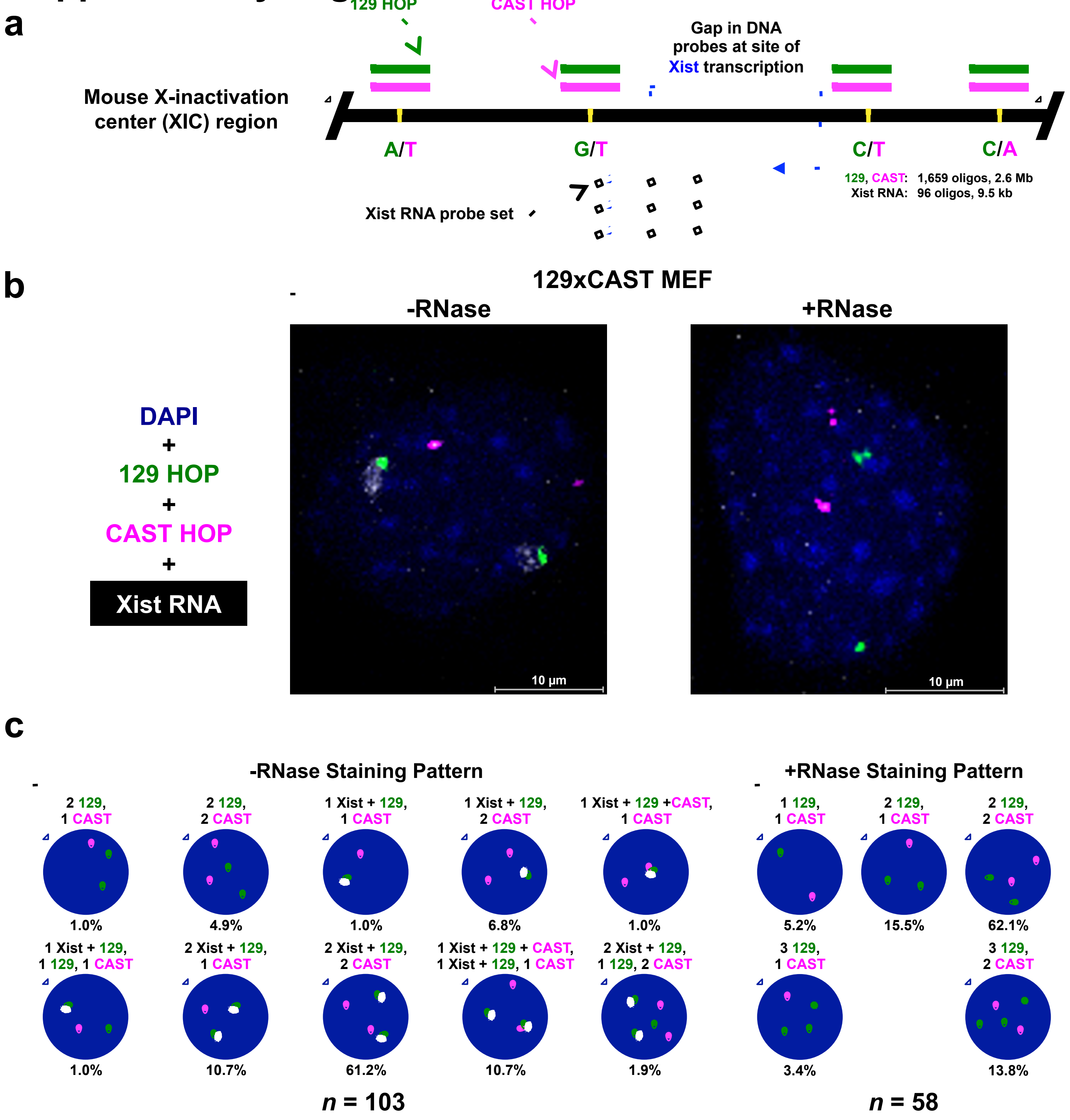

Supplementary Figure 17 RNA/DNA FISH with HOPs. (**a**) Probe sets as in **Fig. 4a**. (**b**) FISH performed with or without the addition of RNase in the hybrid EY.T4 129xCAST line. DNA is stained with DAPI (blue). Images were captured on a laser scanning confocal microscope and are maximum Z projections. (**c**) Staining patterns observed. *n* = nuclei scored.

Supplementary. Figure 18

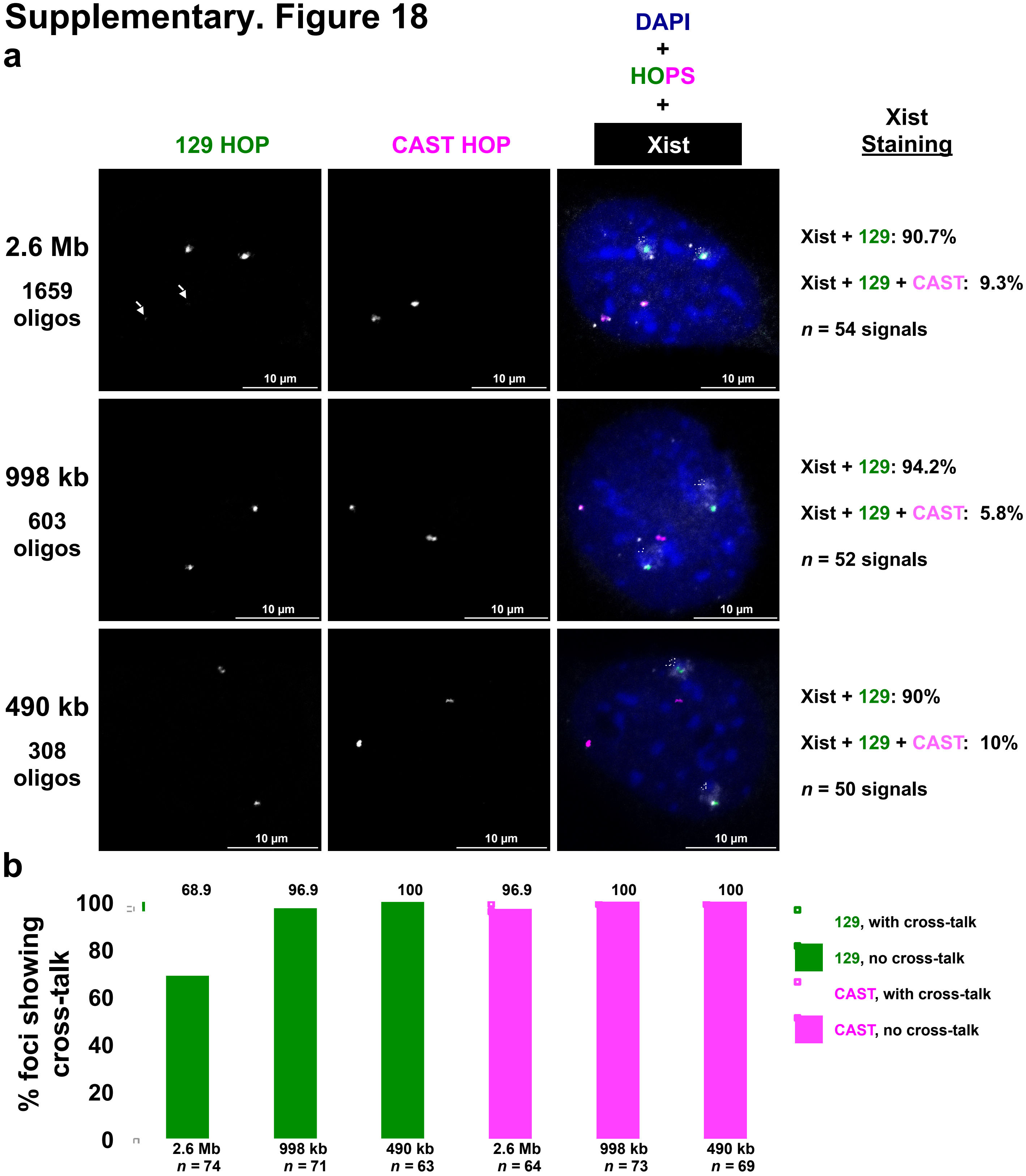

Supplementary Figure 18 FISH with a range of HOP sizes. (**a**) Grayscale and merged images of FISH performed in in the hybrid EY.T4 129xCAST line using the three indicated sets of XIC HOPs. Xist clouds are outlined in white. Images were captured on a laser scanning confocal microscope and are maximum Z projections. The Xist staining pattern for each experiment is summarized in the left column. Arrows indicate cross-talk between the HOPs. (**b**) Analysis of cross-talk between HOP probes for each experiment. Each 129 and CAST focus was scored for the presence (cross-talk) or absence (no cross-talk) of signal in the same position from its cognate HOP. *n* indicates the number of foci scored for each HOP.

Supplementary. Figure 19

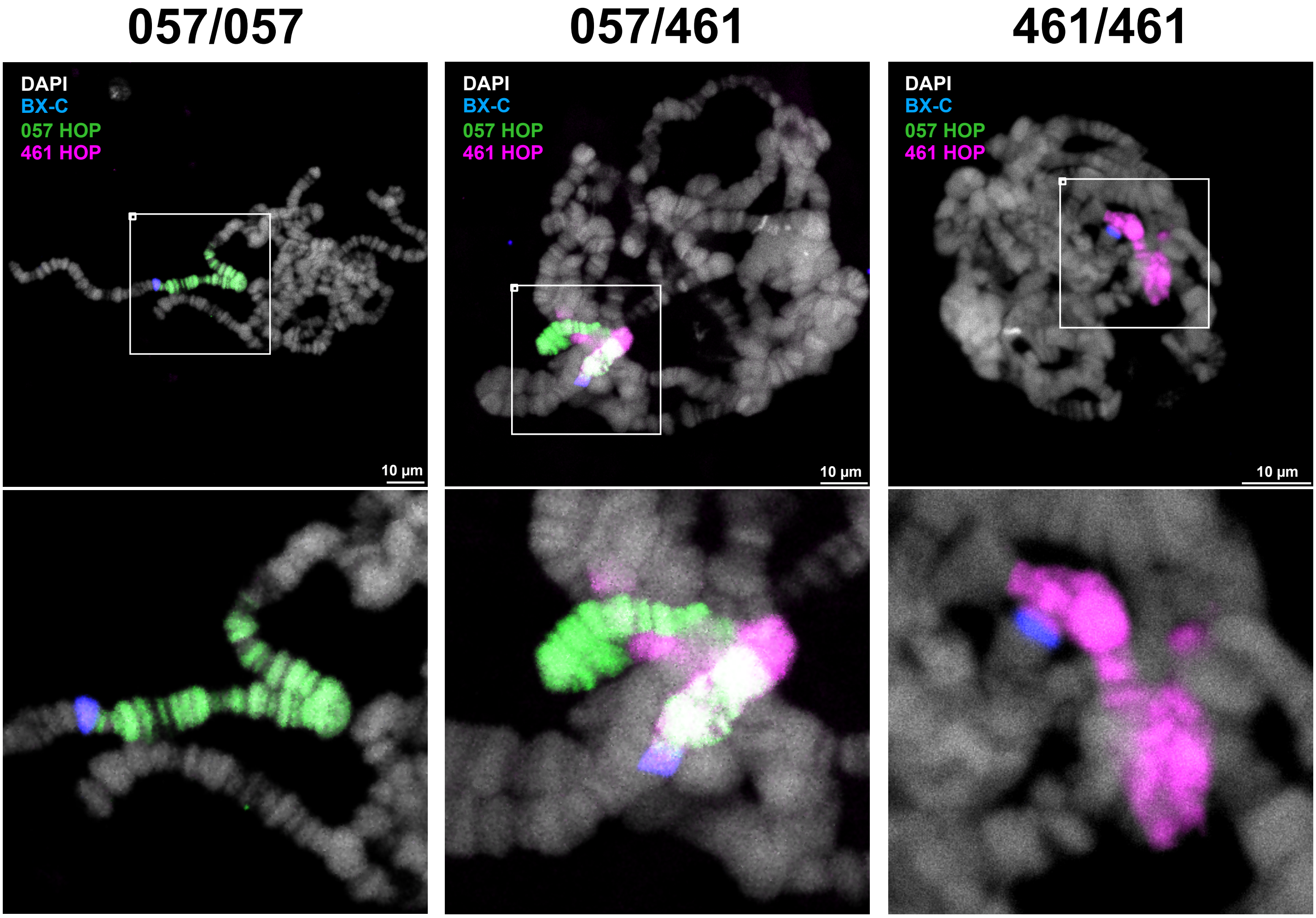

Supplementary Figure 19 HOPs FISH on spread Drosophila polytene chromosomes. FISH was performed on polytene chromosomes isolated from the salivary glands of 057/057 homozygotes (right), 057/461 hybrids (center), and 461/461 homozygotes (left). In all cases, the BX-C probe set (blue) was hybridized to the region adjacent to the 057 (green) and 461 (magenta) 89E-93C HOPs. DNA was stained with DAPI (grey). Bottom panels: zoomed-in images of boxed regions in the corresponding top panels. Images were captured on a laser scanning confocal microscope and are single Z slices. Note that because Drosophila pairs its homologous chromosomes in somatic cells, only one swath of signal is expected per locus targeted, with separation being occasionally observed (e.g. in the center panels).

Supplementary. Figure 20

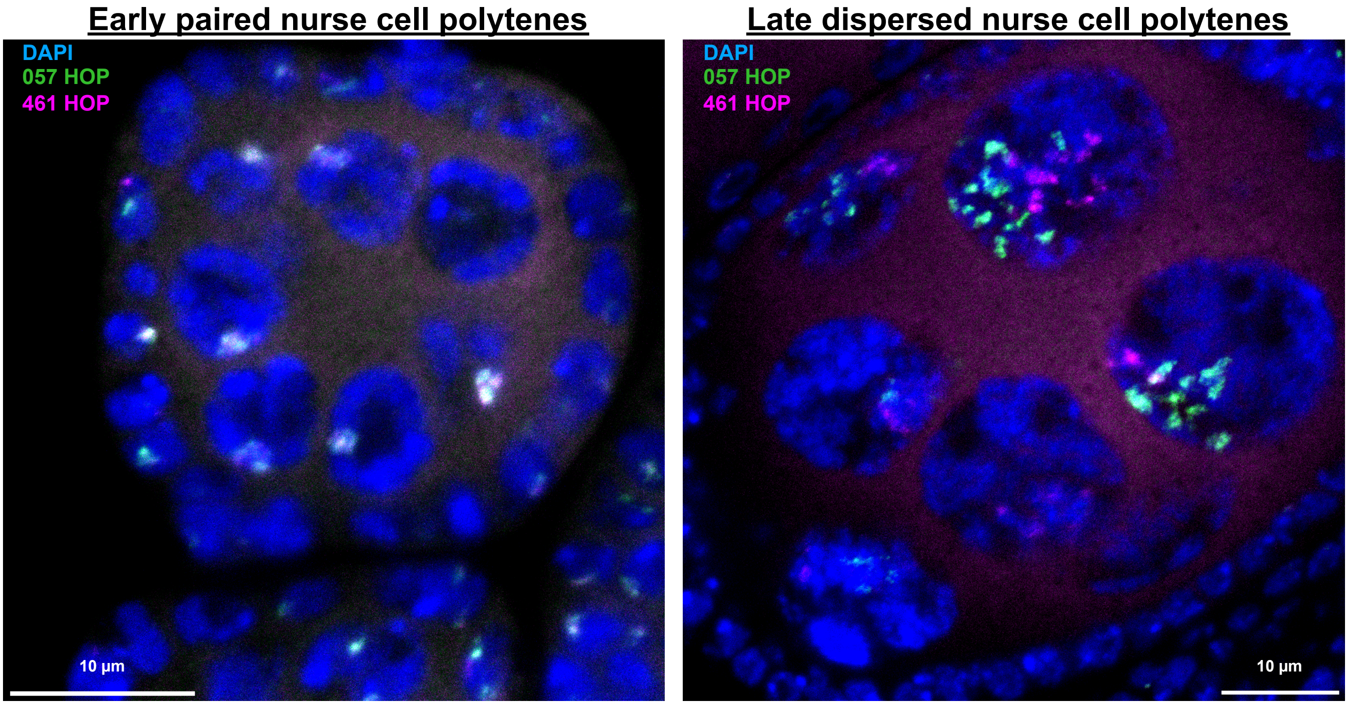

Supplementary Figure 20 HOPs FISH on whole-mount Drosophila ovaries. FISH performed with the 057 (green) and 461 (magenta) 89E-93C HOPs. Left: paired polytenized chromosomes in Stage 2 nurse cells. Right: dispersed polytenized chromosomes in Stage 10 nurse cells. DNA is stained with DAPI (blue). Images were captured on a laser scanning confocal microscope and are single Z slices. For a description of the polytene dispersal phenomenon, please see: Dej KJ & Spradling AC 1999. The endocyte controls nurse cell polytene chromosome structure during Drosophila oogenesis. Development 126(2):293-303.

Supplementary. Table 1

2° oligo	Sequence 5' -> 3'	Binding Sequence 5' -> 3'	%G+C	T_M
1	CACACGCTCTTCCGTTCTATGCGACGTCGGTG	CACCGACGTCGCATAGAACGGAAGAGCGTGTG	59.4	76°C
2	AGAACGATCCAGCGAGATCAAGTGGAGCTGCG	CGCAGCTCCACTTGATCTCGCTGGATCGTTCT	56.2	75°C
3	CATTGCCGTATGGGCTAGGATGACCTGGCTCG	CGAGCCAGGTCATCCTAGCCCATACGGCAATG	59.4	75°C
4	GCATTCACCCTTGCACGATACCGAGCCACACC	GGTGTGGCTCGGTATCGTGCAAGGGTGAATGC	59.4	76°C
5	ACACCCTTGCACGTCGTGGACCTCCTGCGCTA	TAGCGCAGGAGGTCCACGACGTGCAAGGGTGT	62.5	79°C
6	TGATCGACCACGGCCAAGACGGAGAGCGTGTG	CACACGCTCTCCGTCTTGGCCGTGGTCGATCA	62.5	78°C
LNA1[a]	T+CC+AC+GA+CG+TG+CA+A	TTGCACGTCGTGGA	57.1	79°C
LNA2[b]	G+AC+CA+GG+TC+GA+GC+A	TGCTCGACCTGGTC	64.3	80°C
LNA3[c]	C+GA+CT+AG+CA+CC+GG+T	ACCGGTGCTAGTCG	64.3	82°C

Supplementary Table 1 Secondary (2°) oligos and their binding sequences (i.e. the reverse complement of the secondary oligo sequence). For the locked nucleic acid (LNA) oligos, a "+" sign preceding a base indicates an LNA residue at that position. Melting temperature (T_M) values were obtained from Exiqon's online 'LNA Oligo T_M Prediction' tool. [a]Derived from secondary oligo 5. [b]Derived from secondary oligo 6. [c]Derived from secondary oligo 1.

Supplementary. Table 2

Probe set	Hom. Len.	Organism	Assembly	Chr.	Span kb	Start	Stop	Complexity	Probes/kb
27E7-28D3	32	Drosophila	dm3	2L	680	7,256,488	7,936,487	10,000	14.7
89B-89D	42	Drosophila	dm3	3R	176	12,281,443	12,457,345	1,641	9.3
89D-89E/BX-C	42	Drosophila	dm3	3R	316	12,482,502	12,797,965	2,394	7.6
89B 5 kb	36	Drosophila	dm3	3R	4.9	12,274,750	12,279,653	106	21.6
057 HOP	42	Drosophila	dm3	3R	4,201	12,798,329	16,999,743	6,236	1.5
461 HOP	42	Drosophila	dm3	3R	4,201	12,798,329	16,999,743	6,236	1.5
19q13.11-q13.12	32	Human	hg19	19	3,006	33,995,100	37,000,740	20,020	6.7
19q13.2-q13.31	32	Human	hg19	19	2,126	41,281,436	43,407,336	20,020	9.4
19q13.32-q13.33	32	Human	hg19	19	2,267	47,707,413	49,974,862	20,020	8.8
4p16.1	32	Human	hg19	4	51.6	10,168,287	10,219,853	850	16.5
HoxB	42	Mouse	mm9	11	174	96,055,675	96,229,567	1,691	9.7
HoxB 5 kb	36	Mouse	mm9	11	5.0	96,061,359	96,066,320	106	21.2
XIC Interstitial	42	Mouse	mm9	X	2,562	99,370,122	101,932,522	9,058	3.5
129 HOP	42	Mouse	mm9	X	2,562	99,370,785	101,933,168	1,659	0.6
CAST HOP	42	Mouse	mm9	X	2,562	99,370,785	101,933,168	1,659	0.6
129 HOP 998 kb	42	Mouse	mm9	X	998	100,152,309	101,150,694	603	0.6
CAST HOP 998 kb	42	Mouse	mm9	X	998	100,152,309	101,150,694	603	0.6
129 HOP 490 kb	42	Mouse	mm9	X	490	100,411,782	100,901,392	308	0.6
CAST HOP 490 kb	42	Mouse	mm9	X	490	100,411,782	100,901,392	308	0.6
Xist RNA	42	Mouse	mm9	X	9.5	100,669,044	100,678,521	96	10.1

Supplementary Table 2 Oligopaint probe sets used. The length of genomic homology (Hom. Len.), organism targeted, genome assembly, span of the probe set in kilobases, start and stop coordinates of the span, the number of oligos in the probe set (complexity), and the number of probes per kilobase of target are given for each probe set.

Supplementary. Table 3

F primer sequence 5’ -> 3’	R primer sequence 5’ -> 3’	Probes used on
GTATCGTGCAAGGGT<u>GAATGC</u>	GAGCAGTCACAGTCCAGAAGG	89D-89E/BX-C
CGCTCGGTCTCCGTTCGTCTC	GGGCTAGGTACAGGGTTCAGC	129 HOP, 129 998 kb, 057 HOP
CAGGTCGAGCCCTGTAGTACG	CTAGGAGACAGCCTCGGACAC	19q13.32-q13.33, CAST HOP, CAST 990 kb, 461 HOP
GACTGGTACTCGCGTGACTTG	CGTCAGTACAGGGTGTGATGC	89B-89D, CAST 490 kb
GTATCGTGCAAGGGT<u>GAATGC</u>	ATCCTAGCCCATACG<u>GCAATG</u>	89B 5 kb, 19q13.11-q13.12, 19q13.2-q13.31, 4p16.1, Xist RNA, HoxB 5 kb
CCAGTGCTCGTGTGAGAAGTC	CTGCAGAGAAGAGGCAGGTTC	HoxB, XIC Interstitial
CCGAGTCTAGCGTCTCCTCTG	AACAGAGCCAGCCTCTACCTG	129 490 kb

Supplementary Table 3 Primer pairs used. The sequence is given for the forward and reverse primers in each pair, as well as the Oligopaint probe sets that each was used to amplify. Primers that have engineered 3’ nicking endonuclease sites (see Beliveau *et al.* 2012) have the location of the nicking site underlined.

Supplementary. Table 4

Description	Sequence 5' ->3'
2° oligo 1 with 5' label	**/56-FAM/** or **/5Cy3/** or **/5Cy5/** CACACGCTCTTCCGTTCTATGCGACGTCGGTG
2° oligo 2 with 5' label	**/56-FAM/** or **/5Cy3/** or **/5Cy5/** AGAACGATCCAGCGAGATCAAGTGGAGCTGCG
2° oligo 3 with 5' label	**/56-FAM/** or **/5Cy3/** or **/5Cy5/** CATTGCCGTATGGGCTAGGATGACCTGGCTCG
2° oligo 4 with 5' label	**/56-FAM/** or **/5Cy3/** or **/5Cy5/** GCATTCACCCTTGCACGATACCGAGCCACACC
2° oligo 5 with 5' label	**/56-FAM/** or **/5Cy3/** or **/5Cy5/** ACACCCTTGCACGTCGTGGACCTCCTGCGCTA
2° oligo 6 with 5' label	**/56-FAM/** or **/5Cy3/** or **/5Cy5/** TGATCGACCACGGCCAAGACGGAGAGCGTGTG
2° oligo 5 with 3' Cy5	ACACCCTTGCACGTCGTGGACCTCCTGCGCTA**/3Cy5/**
2° oligo 1 2X AlexaFluor 647[a]	**/5Alex647N/**CACACGCTCTTCCGTTCTATGCGACGTCGGTGagatgttt**/3AlexF647N/**
2° oligo 1 2X ATTO 565	**/5ATTO565N/**CACACGCTCTTCCGTTCTATGCGACGTCGGTGtttttttt**/3ATTO565N/**
2° oligo 5 2X AlexaFluor 488[a]	**/5Alex488N/**ACACCCTTGCACGTCGTGGACCTCCTGCGCTAagatgttt**/3AlexF488N/**
2° oligo 6 2X ATTO 633	**/5ATTO633N/**TGATCGACCACGGCCAAGACGGAGAGCGTGTGtttttttt**/3ATTO633N/**

Supplementary Table 4 Secondary oligos used. For each, we give the sequence and position and identity of fluorophores, using the modification codes of Integrated DNA Technologies. Note that the secondary oligos containing both a 5' and 3' label have an eight-base spacer sequence between the 3' end of the secondary oligo and the 3' fluorophore in order to minimize quenching if a 5' fluorophore is present on the primary Oligopaint probe set (see **Supplementary Fig. 11**). [a]These secondary probes represent older designs in which a short orthogonal sequence was used as the spacer. Our newer secondary probes (e.g. Secondary 1 2X ATTO 565) use a poly-T spacer.

Supplementary. Note 1

32mer vs. 36mer and 42mer Oligopaint genomic homology length

Our initial report of Oligopaints (Beliveau *et al.* 2012) focused on oligo probes bearing 32 bases of homology to their genomic target, as this length gave high probe coverage of target loci while remaining compatible with the ~60 base length constraint of oligo arrays at that time. Given the greater lengths of oligos that are now available, we have subsequently explored additional designs for Oligopaints and found that slightly longer lengths of genomic homology give higher probe coverage when a higher T_M threshold is enforced (e.g. minimum T_M = 85°C, vs. 75°C in our initial report), with the higher T_M threshold predicted to allow for more stringent hybridization and wash conditions. While the optimal homology length will likely vary with the %G+C and repeat content of a given genome and target as well as reflect the specific nature of the probe set being designed, we have found 36-42 bases to be a suitable range for the length of genomic homology for Oligopaints targeting Drosophila, mouse, and human genomes.
